# Altered basal forebrain function during whole-brain network activity at pre- and early-plaque stages of Alzheimer’s disease in TgF344-AD rats

**DOI:** 10.1186/s13195-022-01089-2

**Published:** 2022-10-10

**Authors:** Monica van den Berg, Mohit H. Adhikari, Marlies Verschuuren, Isabel Pintelon, Tamara Vasilkovska, Johan Van Audekerke, Stephan Missault, Loran Heymans, Peter Ponsaerts, Winnok H. De Vos, Annemie Van der Linden, Georgios A. Keliris, Marleen Verhoye

**Affiliations:** 1 Bio-Imaging Lab, University of Antwerp, Universiteitsplein 1 2610 Wilrijk, Antwerp, Belgium; 2 µNEURO Research Centre of Excellence, University of Antwerp, Antwerp, Belgium; 3 Laboratory of Cell Biology and Histology, University of Antwerp, Universiteitsplein 1 2610 Wilrijk, Antwerp, Belgium; 4 Antwerp Centre for Advanced Microscopy, Universiteitsplein 1, 2610 Wilrijk, Antwerp, Belgium; 5 Laboratory of Experimental Hematology, Vaccine and Infectious Disease Institute (Vaxinfectio), University of Antwerp, Universiteitsplein 1, 2610 Wilrijk, Antwerp, Belgium; 6 Institute of Computer Science, Foundation for Research & Technology - Hellas, Heraklion, Crete, Greece

**Keywords:** Alzheimer’s disease, Basal forebrain, Resting state functional MRI, Network dysfunction, Gliosis, Synaptic dysfunction, Amyloid, Quasi-periodic patterns

## Abstract

**Background:**

Imbalanced synaptic transmission appears to be an early driver in Alzheimer’s disease (AD) leading to brain network alterations. Early detection of altered synaptic transmission and insight into mechanisms causing early synaptic alterations would be valuable treatment strategies. This study aimed to investigate how whole-brain networks are influenced at pre- and early-plague stages of AD and if these manifestations are associated with concomitant cellular and synaptic deficits.

**Methods:**

To this end, we used an established AD rat model (TgF344-AD) and employed resting state functional MRI and quasi-periodic pattern (QPP) analysis, a method to detect recurrent spatiotemporal motifs of brain activity, in parallel with state-of-the-art immunohistochemistry in selected brain regions.

**Results:**

At the pre-plaque stage, QPPs in TgF344-AD rats showed decreased activity of the basal forebrain (BFB) and the default mode-like network. Histological analyses revealed increased astrocyte abundance restricted to the BFB, in the absence of amyloid plaques, tauopathy, and alterations in a number of cholinergic, gaba-ergic, and glutamatergic synapses. During the early-plaque stage, when mild amyloid-beta (Aβ) accumulation was observed in the cortex and hippocampus, QPPs in the TgF344-AD rats normalized suggesting the activation of compensatory mechanisms during this early disease progression period. Interestingly, astrogliosis observed in the BFB at the pre-plaque stage was absent at the early-plaque stage. Moreover, altered excitatory/inhibitory balance was observed in cortical regions belonging to the default mode-like network. In wild-type rats, at both time points, peak activity in the BFB preceded peak activity in other brain regions—indicating its modulatory role during QPPs. However, this pattern was eliminated in TgF344-AD suggesting that alterations in BFB-directed neuromodulation have a pronounced impact in network function in AD.

**Conclusions:**

This study demonstrates the value of rsfMRI and advanced network analysis methods to detect early alterations in BFB function in AD, which could aid early diagnosis and intervention in AD. Restoring the global synaptic transmission, possibly by modulating astrogliosis in the BFB, might be a promising therapeutic strategy to restore brain network function and delay the onset of symptoms in AD.

**Supplementary Information:**

The online version contains supplementary material available at 10.1186/s13195-022-01089-2.

## Background

Alzheimer’s disease (AD) is a severe neurodegenerative disorder with an insidious onset of disease. Due to an aging society, an increasing number of people are expected to suffer from this incurable disorder, bringing enormous economic and societal burdens. Despite decades of research and numerous clinical trials, disease-modifying therapies are still lacking, mainly due to the incomplete insight into the pathological mechanisms of AD [1]. AD is characterized by a progressive accumulation of two toxic proteins, the amyloid-beta (Aβ) and hyperphosphorylated tau (pTau), inducing neuronal loss, cognitive impairment, and dementia. Since the accumulation of Aβ is one of the earliest events in AD pathology, it has long been hypothesized that it triggers the formation of pTau and neurodegeneration. However, Aβ plaque accumulation is not linearly correlated with dementia symptoms. Interestingly, neuronal hyperexcitability (resulting from increased glutamatergic and decreased GABA-ergic signaling) has been observed prior to plaque formation [2–5]. This hyperexcitability exacerbates the formation of soluble Aβ species, therefore driving disease progression [5]. Soluble monomeric and oligomeric Aẞ have been demonstrated to be neurotoxic to cholinergic neurons originating from the basal forebrain (BFB), causing the BFB to become affected during early stages of AD [6]. Studies have demonstrated that decreased BFB volume precedes the cortical spread of Aẞ [2, 3]. The BFB has been implicated in the modulation of cognition, specifically the projections from the diagonal band of Broca, substantia innominata, and the nucleus basalis of Meynert (NBM) to the cortex [4, 5]. Moreover, the BFB is known to be an important modulator of network activity and is involved in cortical network switching between brain states by exerting direct and/or indirect control on the prefrontal cortex [7–9]. Therefore, alterations in BFB signaling due to AD pathology might lead to early disruption of network activity in the brain.

Resting state functional MRI (rsfMRI) is a non-invasive MRI technique, which uses the blood oxygen level-dependent (BOLD) contrast to indirectly assess spontaneous neuronal activity while the subject is at rest. Low-frequency, temporally correlated fluctuations of BOLD activity between brain regions have been shown to consistently exist across certain distally located areas that are defined as resting state networks [10–12]. Moreover, the strength of the temporal correlation is thought to be representing functional connectivity (FC) between the nodes of these networks. We recently demonstrated that activation of cholinergic neurons in the NBM induces a decrease of FC in the default mode-like network (DMLN) [13], a major resting state network active during self-referential tasks such as mind wandering and introspection [14]. Numerous clinical rsfMRI studies have detected FC alterations in patients with mild cognitive impairment and AD-related dementia, as well as in animal models of AD. Aberrations in FC were mainly observed in the default mode network and have been linked to cognitive and behavioral performance setting it as an important target for therapies [15–21].

Recent advances in dynamic rsfMRI analysis approaches revealed fundamental new insights regarding regional involvement, and regulation of so-called brain states, by considering the dynamic, temporal properties of FC [22–24]. In addition, other dynamic analysis methods—related but not identical to FC—have also been proposed and offer some important advantages such as increased time resolution and a better estimation of the instantaneous or short-term relationship of activity across areas [25–28]. One such method is the quasi-periodic pattern (QPP) analysis, which detects recurrent, relatively short-lived spatiotemporal motifs of whole-brain activity using the BOLD signal. One of the strengths of QPPs is that they are a sensitive method to directly demonstrate the anti-correlated activity between the default mode-like network, and the lateral cortical network (LCN) in rodents [29]—the rodent analog of the so-called task-positive network in humans, a network that has been shown to be activated in cognitive and attention demanding tasks [12]. Thus, we speculated that QPP analysis would be very powerful and could offer novel insights into spatial and temporal alterations in neuronal activity during specific recurrent brain states at pre-plaque stages of AD. Moreover, QPP analysis could potentially uncover regions involved in the modulation and regulation of these states and networks.

Imbalance in synaptic function appears to be an early driver of AD progression [30–34]. Thus, sensitive measures of early network disturbances could assist in the development of promising biomarkers for early diagnosis of AD. Moreover, targeting the impaired network function during this pre-symptomatic phase might prove imperative for disease-modifying treatments. However, the mechanisms underlying the early network dysfunction are still unknown. In this study, we hypothesized that during the pre-plaque stage, alterations in BFB activity induce changes in whole-brain, recurrent patterns of brain activity involving the DMLN and LCN, which can be detected with rsfMRI and QPP analysis. We test this hypothesis using rsfMRI in a well-characterized, highly translational TgF344-AD rat model of AD, which bears human APP_swe_ and PS1_ΔE9_ mutations, resulting in an age-dependent accumulation of Aβ depositions [35], endogenous pTau accumulation [36, 37], and cognitive deficits, akin to human AD [38]. Furthermore, to unravel the concomitant cellular and synaptic deficits underlying network alterations, QPP analysis was complemented with the state-of-the-art histological investigation of relevant brain areas such as BFB and nodes of the DMLN and LCN.

## Material and methods

### Animals and ethical statement

TgF344-AD and F344 rats for this study were bred in-house. Heterozygous TgF344-AD rats were purchased from the RRRC (RRID:RGD_10401208) and were coupled with F344 rats (Charles River, Italy). The male offspring was used in this study. Fifteen male TgF344-AD rats and 11 wild-type (WT) littermates underwent rsfMRI scans at both 4 and 6 months of age. An additional 42 male rats were used for histological (6 WT and 6 TgF344-AD rats for both time points) and biochemical analyses (3 WT and 6 TgF344-AD rats for both time points). Animals were group housed with a 12-h light/dark cycle (lights on at 7 am) and with controlled temperature (20–24 °C) and humidity (40–60%) conditions. Standard food and water were provided ad libitum. All procedures were in accordance with the guidelines approved by the European Ethics Committee (decree 2010/63/EU) and were approved by the Committee on Animal Care and Use at the University of Antwerp, Belgium (approval number: 2019–06).

### In vivo* MRI procedures*

Rats were anesthetized using isoflurane (5% for induction and 2% during handling procedures). The animals were endotracheally intubated and mechanically ventilated (70 breaths per minute) using a ventilator (Microventilator, Carfil, Belgium) under 2% isoflurane. Ear bars were used to fix the head of the animal and a cannula was placed in the tail vein, after which an intravenous bolus injection of medetomidine (0.05 mg/kg, Domitor®, Pfizer, Germany) and pancuroniumbromide (0.5 mg/kg, VWR, Belgium) was administered. The constant intravenous infusion of medetomidine (0.1 mg/kg/h) and pancuroniumbromide (0.5 mg/kg/h) was started 15 min after the administration of the bolus and the isoflurane concentration was gradually lowered to 0.4%. Animal physiology was closely monitored during the handling and the scanning. Body temperature was maintained at 37 ± 0.5 °C using a feedback-controlled warm air circuitry (MR-compatible Small Animal Heating System, SA Instruments, Inc., USA). A pressure-sensitive pad (sampling rate 225 Hz; MR-compatible Small Animal Monitoring and Gating system, SA Instruments, Inc., USA) was used to record the breathing rate. Additionally, a pulse oximeter was placed on the hind paw of the animal to monitor the heart rate and blood oxygenation (MR-compatible Small Animal Monitoring and Gating System, SA Instruments, Inc., USA).

#### MRI acquisition

Data were acquired using a 9.4 T Biospec MRI system (Bruker BioSpin, Germany) with Paravision 6 software, using a 2 × 2 array receiver head radiofrequency coil. To allow uniform slice positioning, T2-weighted TurboRARE images were acquired along three directions (TR 2500 ms, TE 33 ms, FOV (30 × 30) mm^2^, matrix [128 × 128]). Magnetic field inhomogeneity was corrected by local shimming in an ellipsoid volume of interest covering the brain. Resting state functional MRI (rsfMRI) was acquired using a single shot gradient echo EPI-sequence (TR 600 ms, TE 18 ms, FOV (30 × 30) mm^2^, matrix [96 × 96], 12 coronal slices of 1.0 mm, slice gap 0.1 mm, 1000 repetitions). RsfMRI scans started 35 min after the initial bolus administration and the total scan time was 10 min. Intravenous infusion of medetomidine and pancuroniumbromide anesthesia was terminated after completion of the rsfMRI scan and isoflurane was increased to 2%. Next, T2-weighted 3D images were acquired using a 3D RARE sequence (TR 2500 ms, effective TE 44 ms, FOV (30 × 30x22) mm^3^, matrix [256 × 256 × 128], RARE-factor 16). At the end of the scan session, a subcutaneous injection of 0.1 mg/kg atipamezole (Antisedan®, Pfizer, Germany) was administered to counteract the effects of the medetomidine anesthesia and the animals were put on a second ventilator and heating pad to recover. All animals recovered within 1 h after the end of the scan session, except for two 4-month-old TgF344-AD rats that did not recover after the scan session due to premature extubation.

#### Data preprocessing

Preprocessing of the data including debiasing, normalization, realignment, smoothing, and co-registration was performed using SPM12 software (Statistical Parametric Mapping). First, we performed debiasing on the 3D RARE scans in order to remove the smooth, spatially varying intensity gradient induced by the receiving RF coil. Next, we created a study-specific 3D template from a subset (4 M 11 WT/15 TG–6 M 11 WT/13Tg) of animals debiased 3D RARE scans in Advanced Normalization Tools (ANTS). Then, all rsfMRI images were realigned to the first image using a 6-parameter rigid body spatial transformation estimated using a least-square approach. Next, rsfMRI images were coregistered to the anatomical 3D scan of the same imaging session using a global 12-parameter affine transformation with mutual information as the similarity metric. The anatomical 3D scan was finally spatially normalized to the study-specific 3D template using a global 12-parameter affine transformation followed by a non-linear deformation protocol. The combined transformation (realignment and spatial normalization to the template) is applied to realign all EPI and co-register them to the 3D RARE template. Finally, ventricles were masked out of the data and in-plane smoothing was performed using a Gaussian kernel with full width at half maximum of twice the voxel size.

#### Quasi-periodic pattern (QPP) analysis

Global signal regression, quadratic detrending, demeaning, and Fischer *z*-score transformation were performed on preprocessed rsfMRI scans, after which the scans were filtered between 0.01 and 0.17 Hz using a Butterworth band pass filter. Next, rsfMRI scans were concatenated per group and per time point to create an image series. QPPs were extracted using a modified algorithm first described by [39], which detects recurrent, spatiotemporal patterns of BOLD activity. The first step in this algorithm extracts a template, which is a set of a pre-specified number (i.e., window size) of consecutive BOLD images, derived at a randomly selected seed time frame within the image series. This template is then incrementally shifted along the image series, and the spatial correlation of the BOLD signals, restricted to a brain mask, of the template and the successive image series segments of the same is calculated. The resulting correlation time series is termed the sliding template correlation (STC). Local peaks in the STC with peak correlation exceeding a threshold of 0.2 are identified and the corresponding image series segments are averaged, voxel-wise, to form an updated template. This process is repeated until the spatial correlation between the updated and previous template is at least 0.99 resulting in a QPP. Thereafter, the whole process is repeated with a new random seed location. The length of the template and hence the resulting QPP are defined by the user prior to the extraction. The number of QPPs extracted is based on the number of chosen random seed points. In this study, 200 QPPs were identified with a length of 6 TR (3.6 s) and 15 TR (9 s).

For both short and long window sizes, hierarchical clustering was performed based on temporal and spatial similarity to identify clusters of spatiotemporal similar QPPs. Robust clusters were identified using several criteria including a minimum cluster size of 20 QPPs, > 10 occurrences across the whole image series, and the majority of the QPPs of that cluster occur, at least once, in at least 80% of the subjects. The QPPs with the highest occurrence within their cluster, as measured by the instances the STC exceeds the threshold of 0.2, were selected as representative QPPs (rQPPs).

To visualize and identify the sequential regional activation and deactivation pattern that characterize a specific rQPPs, one-sample *t*-tests (two-tailed, FDR *p* < 0.05, minimum cluster size 10 voxels, Matlab) were performed on the BOLD signals of the instances the STC exceeds the threshold of 0.2. QPP clusters were matched between groups using a spatial cross-correlation coefficient after which the activation patterns of spatially matched rQPPs (Pearson correlation coefficient > 0.70) were compared using a two-sample *t*-test (two-tailed, FDR *p* < 0.05, minimum cluster size 10 voxels, Matlab) and the effect size was estimated by calculating the Cohens *d* on the ROI-based time courses for each time point in the QPP separately. Each QPP has its own STC from the image series they are derived from, which describes the similarity between the QPP and the image series, and which can be used to extract occurrence rates at the subject level. Cluster-wise occurrence rates of matched clusters were compared between groups using an unpaired two-sample *t*-test (two-tailed, FDR *p* < 0.05, Matlab).

To evaluate the propagation of activity during QPPs, 9-s QPPs were extracted and representative QPPs were selected based on hierarchical clustering as described above. Representative QPPs of the TgF344-AD rats were phase-aligned to the QPP of the WT animals based on the maximal spatial correlation between the rQPPs. To evaluate BOLD activity during QPPs in specific regions of interest (ROI), group-averaged BOLD time courses from each ROI were extracted from the image series and plotted. Next, the peak timings of the (non-*z*-scored) BOLD signal within each voxel in a ROI were averaged across all QPP occurrences over the group, to create a voxel-wise distribution of mean peak timings within each ROI. Unpaired two-sample *t*-tests (FDR *p* < 0.05, Matlab) were performed to compare mean, across ROI voxels, peak timings between regions within each group in order to identify significant differences in the timing of peak activity of regions.

### Immunohistochemistry

#### Immunofluorescent stainings

To evaluate AD pathology and alterations in synaptic markers, histological analyses were performed on cryosections of 12 TgF344-AD rats (4 months N = 6, 6 months N = 6) and 12 WT littermates (4 months N = 6, 6 months N = 6). The rats were deeply anesthetized using an intravenous injection of pentobarbital (Dolethal®, Vetoquinol, Belgium). Cardiac perfusion was performed with an ice-cold PBS solution, followed by a 4% paraformaldehyde solution (Merck Millipore, Merck KGaA, Darmstadt, Germany) for fixation of the tissues. Brains were surgically removed and post-fixed in 4% paraformaldehyde solution after which a sucrose gradient (5%, 10%, and 20%) was applied. Brains were snap frozen in liquid nitrogen and stored at − 80 °C until further processing. For immunohistochemistry, hemispheres were separated and left hemispheres were embedded in an OCT-embedding medium for sectioning. At Bregma levels 0.4, 1.40, and 3.90, sagittal sections of 12 µm were made using a Leica CM1950 cryomicrotome (Leica BioSystems, Belgium), thaw-mounted on VWR Superfrost Plus micro slides (VWR, Leuven, Belgium) and dried for 2 h at 37 °C.

All immunohistochemical incubations were carried out at RT, using non-consecutive sections per Bregma level and staining. Primary and secondary antibodies were diluted in a blocking buffer containing 0.01 M PBS, 10% normal horse serum, 0.01% bovine serum albumin, 0.05% thimerosal, and 0.01% sodium azide. In short, sections were preincubated for 30 min in a blocking buffer containing 1% Triton X-100 before an overnight incubation with the primary antibodies. Glial cells were visualized with a combination of GFAP (1:200, Goat, Abcam, Ab53554, RRID:AB_880202) and Iba1 (1:500, Rabbit, Fujifilm Wako Chemicals, 019–19,741, RRID:AB_839504). To investigate the synaptic excitatory/inhibitory balance, a triple staining was performed using VGAT (1:200, Rabbit, Synaptic Systems, 131,003, RRID:AB_887869); VGLUT (Guinea Pig, Synaptic Systems, 135,304, RRID:AB_887878); VAChT (1:100, Goat, Merck, ABN100, RRID:AB_2630394). For the detection of the immunoreactivity, the sections were incubated for 4 h with the appropriate combination of fluorescent-conjugated secondary antibodies (Jackson Immunoresearch, West Grove, USA). Followed by a nuclear counterstain using 4′,6-diamidino-2-phenylindole (DAPI, Sigma-Aldrich, Hoeilaart, Belgium) for 10 min at room temperature. Samples were mounted in Citifluor AF1 (EMS, Hathfield, USA) or the permanent Citifluor PVP-1 + Antifadent (EMS) for whole slide imaging. Respective single-labeling studies which resulted in comparable staining were performed to rule out nonspecific findings resulting from the multiple-staining process. Negative staining controls were performed by substitution of non-immune sera for the primary or secondary antisera.

To follow the progression of the AD Aβ plaques and pTau were visualized. X-34 was used as pan-Aβ marker and pTau was specifically detected using a mouse AT8 antibody (1:500, pSer202/Thr205/PSer208, Invitrogen, MN1020, RRID:AB)223,647). This antibody was labeled with a near-infrared fluorescent tag (PerkinElmer VivoTag 680XL) following the manufacturer’s protocol. Sections were permeabilized for 15 min with 0.02% Triton in 0.01 M PBS prior to a 20-min incubation with x-34 (10 µM) in 40% ethanol. In a next step, sections were further incubated for 30 min with a blocking buffer containing 1% Triton X-100 followed by an overnight incubation with AT8 antibody diluted in the blocking buffer. Propidium iodide (5 µg/mL; 5 min; Sigma-Aldrich) was used as a nuclear stain. Sections were mounted using a permanent mounting medium Citifluor PVP-1 + Antifadent.

#### Image acquisition

To acquire images of fluorescently stained sections, a Zeiss Axioscan Z1 (Zeiss, Leuven, Belgium) was used with a Zeiss Colibri 7 solid-state light source of which 385 nm, 475 nm, 555 nm, and 630 nm were used, a filter set 90 HE LED suitable for the applied fluorescent dyes (DAPI, FITC, Cy3, and Cy5), and a Hamamatsu Orca Flash 4.0 V3 digital camera. Zeiss Zen software was used to control the image acquisition and stitching. Whole slide scanning was done with a10 × Plan-apo objective (NA 0.45).

Confocal images of immunolabeled tissue sections were acquired with a Perkin Elmer Ultraview Vox dual spinning disk confocal microscope, mounted on a Nikon Ti body using a 40 × Plan Apo objective (NA 0.95). Lasers with wavelengths 405 nm, 488 nm, 561 nm, and 640 nm were used in combination with a quadruple dichroic and 445/60–525/50–615/70–705/90 nm emission filters. Detection was done on a Hamamatsu C9100-50 CMOS camera. Image acquisition was done using Volocity software. Regions of interest were localized based on the DAPI staining. Per animal, 3 images were acquired on 3 non-consecutive sections in 3 axial positions separated by a 2-μm spacing.

#### Image analysis

Image analysis for neuroinflammation and Aß and pTau pathology was done using the free, open-access image analysis software QuPath (v0.3.0) [40]. On whole section images, the regions of interest were annotated manually based on the nuclear counterstain. For each marker, an intensity threshold was manually defined to measure the positive area percentage with respect to the annotated region. For each marker, the threshold settings were kept constant for all regions and ages studied.

Image analysis of the synaptic excitatory/inhibitory ratio was done in FIJI image analysis software [41]. A macro script was written for FIJI image analysis software [1] to detect synaptic markers and measure their intensity and is available on github (https://github.com/DeVosLab/SynapseDetection). This script is built on modules that were already integrated in similar image analysis pipelines previously developed [42, 43]. After the maximum projection of the Z-stacks, synaptic marker spots were enhanced using a single or multi-scale Laplace filter with user-defined kernel sizes. For each marker, the threshold settings were kept constant for both groups and ages studied. A manually defined threshold per region was applied to segment the spots after which an additional max finding (and region growing) step was included to untangle clustered spots. Only spots that had a projected area within a specific range (0.20–3.00 µm) were retained. Images with more than 10,000 spots were discarded to exclude over-segmented images. During manual verification of images, it was noticed that cholinergic markers labeled cell bodies in MS, HDB/SI, and NBM regions. The cell bodies were excluded from the search region to avoid the detection of spots within these cell bodies. Detection of cell bodies was done after applying a median filter with a large user-defined kernel size, followed by a user-defined threshold and detection of objects larger than 20 µm.

### ELISA

TgF344-AD rats (4 months N = 6, 6 months N = 6) and WT littermates (4 months N = 3, 6 months N = 3) were deeply anesthetized with an intravenous injection of pentobarbital (Dolethal®, Vetoquinol, Belgium) after which cardiac perfusion with an ice-cold PBS solution was performed. The brains were surgically removed, separated by hemisphere, and snapshot frozen in liquid nitrogen. Both hemispheres were stored at − 80 °C until further processing.

Next, Aβ was extracted using an adapted protocol first described by Izco et al. [44]. Briefly, right hemispheres were homogenized (100 mg tissue/mL) on ice using Tris-buffered saline (TBS, Sigma) supplemented with protease inhibitors (HALT, Invitrogen, ThermoFisher Scientific, Belgium). After two short blasts of sonication (30 s), homogenates were centrifuged for 90 min at 20,800 g. Supernatans, which contained the TBS soluble fraction of amyloid monomers and oligomers, were collected, aliquoted, and stored at − 80 °C for further analysis.

Sandwich-ELISA assays were performed according to the manufacturer’s guidelines, to detect monomeric Aβ 1–40 (Invitrogen, KHB3481, ThermoFisher Scientific, Belgium) and Aβ 1–42 (Invitrogen, KHB3441, ThermoFisher Scientific, Belgium) and oligomeric Aβ species (Invitrogen, KHB3491, ThermoFisher Scientific, Belgium). All kits were sensitive to human recombinant AB species. The ELISA signal was quantified using a microplate reader (Versamax, GE Healthcare, Belgium).

### Statistics

Regarding the pathological stainings (Aβ plaques and pTau) and neuroinflammatory stainings (astrogliosis and microgliosis), outliers were detected per genotype, age, and region, based on principal component analysis. Measurements with a *T*^2^ statistics indices higher than the 95% confidence interval were excluded. Two-way ANOVA (genotype, age, genotype*age) per region was performed to evaluate differences in %Area in p-tau, astrogliosis, and microgliosis. In case of a significant interaction, post hoc tests were performed using Student’s *t*-test with FDR correction (*p* < 0.05). For the analysis of the synaptic markers, outlier detection was performed using the interquartile range (1.5 × IQR) per genotype, age, and region on all individual images (3 per animal). Animals with less than 2 images were excluded and the remaining images were averaged for each animal. A second outlier detection was performed on the subject averages, using PCA. Two-way ANOVAs (genotype, age, genotype*age) per region were used to evaluate differences in the vGlut/vGAT ratio and the number of cholinergic synapses, followed by a post hoc FDR-corrected Student’s *t*-test (FDR *p* < 0.05). All statistical analyses were performed in JMP Pro 16. For statistical analysis of the ELISA data, both wild-type groups were combined, since no significant differences between ages were observed in this group. Protein concentrations of all animals were normalized to the mean concentration of the WT. Next, Mann–Whitney *U* tests (Bonferroni corrected) were performed between groups, using Graphpad Prism 9.0.

## Results

### Histological evaluation of AD pathology in 4-month-old TgF344-AD rats

**Fig. 1:**
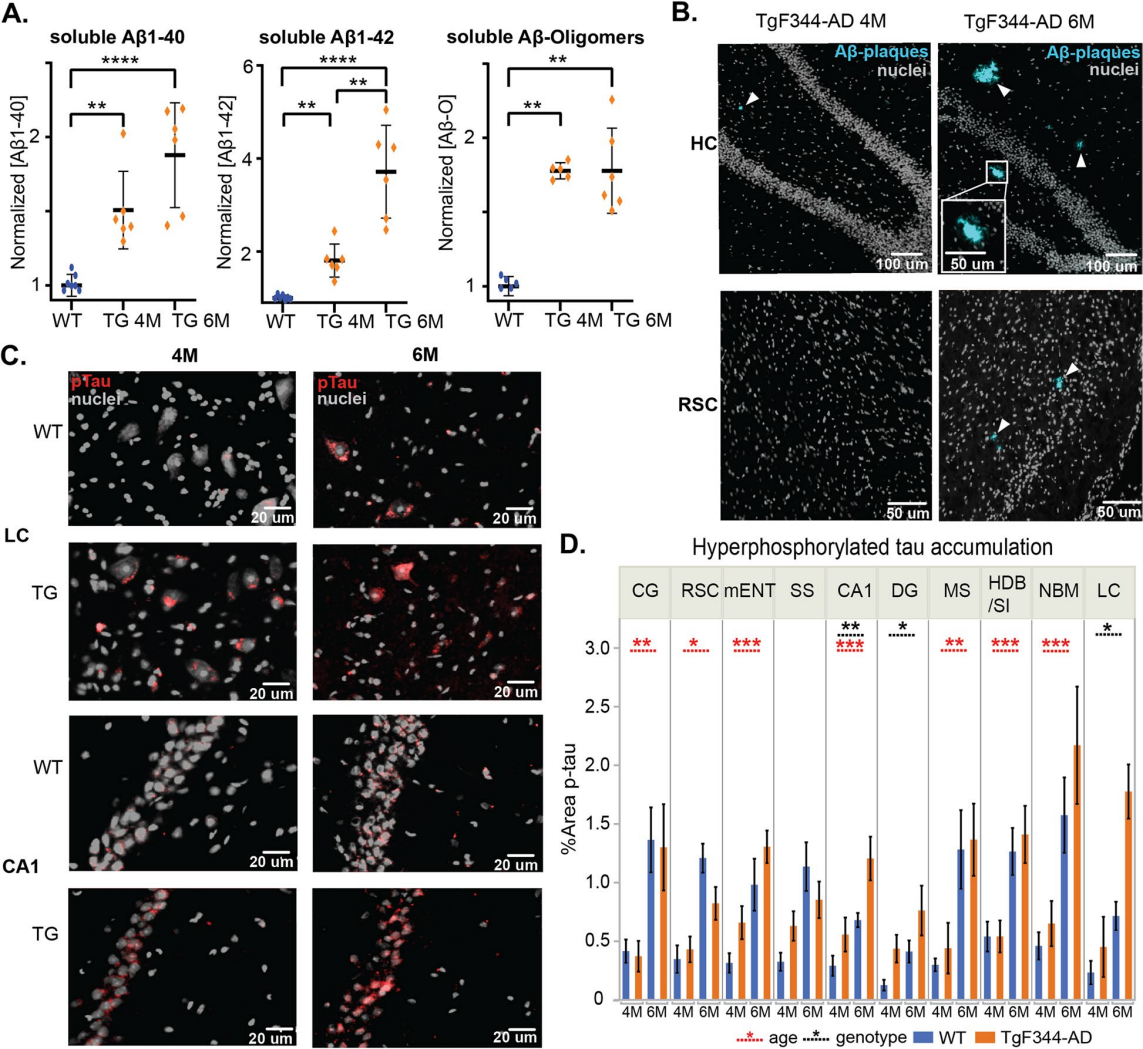
Aβ pathology and pTau pathology in TgF344-AD rats. **A** Biochemical analysis of soluble Aβ monomers and oligomers. Graphs show the mean ± SEM. The marks show the concentrations of each individual animal; individual values were normalized to the mean values of the WT across both ages. Mann–Whitney *U* tests with Bonferroni correction for multiple comparisons were performed to evaluate statistical differences in concentrations. **B** Representative histological images of Aβ plaques (light blue—arrowheads) in TgF344-AD rats. Nuclei are counterstained in gray. **C** Representative images of pTau accumulation (red) in the locus coeruleus (LC) and CA1 region of the hippocampus (CA1). Nuclei are counterstained in gray. **D** Graphs represent the percentage area positive for pTau for 10 different brain regions (mean ± SEM). The blue color represents the wild-type (WT) animals, while the orange represents the TgF344-rats. Dashed lines indicate significant genotype effects (black) or age effects (red) from 2-way ANOVA. *p < 0.05, ** *p* < 0.01, *** *p* < 0.001, **** *p* < 0.0001. CG cingulate cortex, RSC retrosplenial cortex, mENT medial entorhinal cortex, SS somatosensory cortex, MS medial septum, DG dentate gyrus, LC locus coeruleus, HDB/SI horizontal limb of the diagonal band of Broca/substantia innominata, NBM nucleus basalis of Meynert

Previous histological analysis in the TgF344-AD rat model demonstrated an age-dependent development of Aẞ pathology and tauopathy. From 6 months onwards, Aβ plaques and pTau accumulations were observed in the TgF344-AD rats [35, 36]. The goal of this study was to investigate how network activity was altered before Aẞ plaques were present in the brain, during the so-called pre-plaque stages of AD. Therefore, immunohistochemical and biochemical analyses were performed in 4-month-old, our assumed pre-plaque stage, and 6-month-old TgF344-AD rats. Soluble Aẞ species were investigated by performing ELISA immune-assays on brain homogenates of complete hemispheres. Four-month-old TgF344-AD rats demonstrate significantly increased concentrations of TBS soluble Aβ monomers, Aβ1-40 (*p* = 0.0021), Aβ1-42 (*p* = 0.0021), and oligomers (Aβ-o) (*p* = 0.0129) compared to WT (Fig. 1A). While the concentrations of Aβ-o and Aβ1-40 remained at the same concentrations in 6-month-old TgF344-AD rats (Aβ-o; *p* = 0.5368, Aβ1-40; *p* = 0.0931), the concentrations of Aβ1-42 significantly increased in TgF344-AD rats from 4 to 6 months of age (*p* = 0.0022). Immunohistochemistry (IHC) with a pan-Aβ marker X-34 demonstrated the absence of amyloid plaques in the cortex, hippocampus, and BFB in 4-month-old TgF344-AD rats, except one animal with a small plaque present in the hippocampus. At 6 months of age, accumulation of Aβ plaques was observed in the hippocampus and cortex in all TgF344-AD rats (Fig. 1B, Suppl. Figure 1). Immunohistochemical analysis of AT8-stained pTau accumulations revealed a significant effect of age in the cingulate cortex (CG), horizontal limb of the diagonal band of Broca and substantia innominata (HDB/SI), medial septum (MS), entorhinal cortex (mENT), nucleus basalis of Meynert (NBM), and retrosplenial cortex (RSC) with increased pTau in both WT and TgF344-Ad rats at 6 months of age compared with 4 months (Fig. 1C, D). A significant genotype effect was observed in the locus coeruleus (LC), demonstrating increased pTau accumulation in the TgF344-AD rats. In addition, a significant genotype effect was observed in the dentate gyrus (DG), showing an increased pTau accumulation in the TgF3440AD rats, whereas a significant effect of age and genotype was observed in the CA1 region of the hippocampus, both showing an increased pTau accumulation over time and increased pTau in the TgF344-AD rats compared to WT (Suppl. Table 1). Aforementioned results indicate that the 4-month-old time point is a pre-plaque stage in TgF344-AD rats, while the 6-month-old time point resembles the early-plaque stage.

### Quasi-periodic pattern analysis in 4-month-old TgF344-AD rats and WT littermates

The great majority of studies investigating network aberrations in AD employed classical FC analysis. Thus, as a first and reference estimate of network aberrations in our model, we performed a region of interest (ROI)-based analysis starting at 4-month-old TgF344-AD rats and littermate WT controls. Selected ROIs were important functional nodes of the default mode-like network (DMLN), hippocampal network (Hipp), lateral cortical network (LCN), sensory cortical network (Sens), and subcortical network excluding the hippocampal network and were based on the F344 atlas [45]. In Supplementary Fig. 2, the FC matrix including the group-averaged *z*-scored FC between each ROI pair is presented. In both groups and at both ages, FC can be observed within the LCN and between regions belonging to the DMLN, Hip, and Sens networks. In the WT animals, anti-correlation can be observed between these networks and the LCN as is demonstrated by the blue color of the connections between these regions. This anti-correlation has a lower correlation value in the TgF344-AD rats at 4 months of age. However, two-sample statistical tests revealed no significant differences between genotypes after FDR correction, suggesting that static FC was not altered at the pre- and early-plaque stages of AD in the TgF344-AD rats (Suppl. Figure 2, Suppl. Figure 3, Suppl. Table 2).

**Fig. 2:**
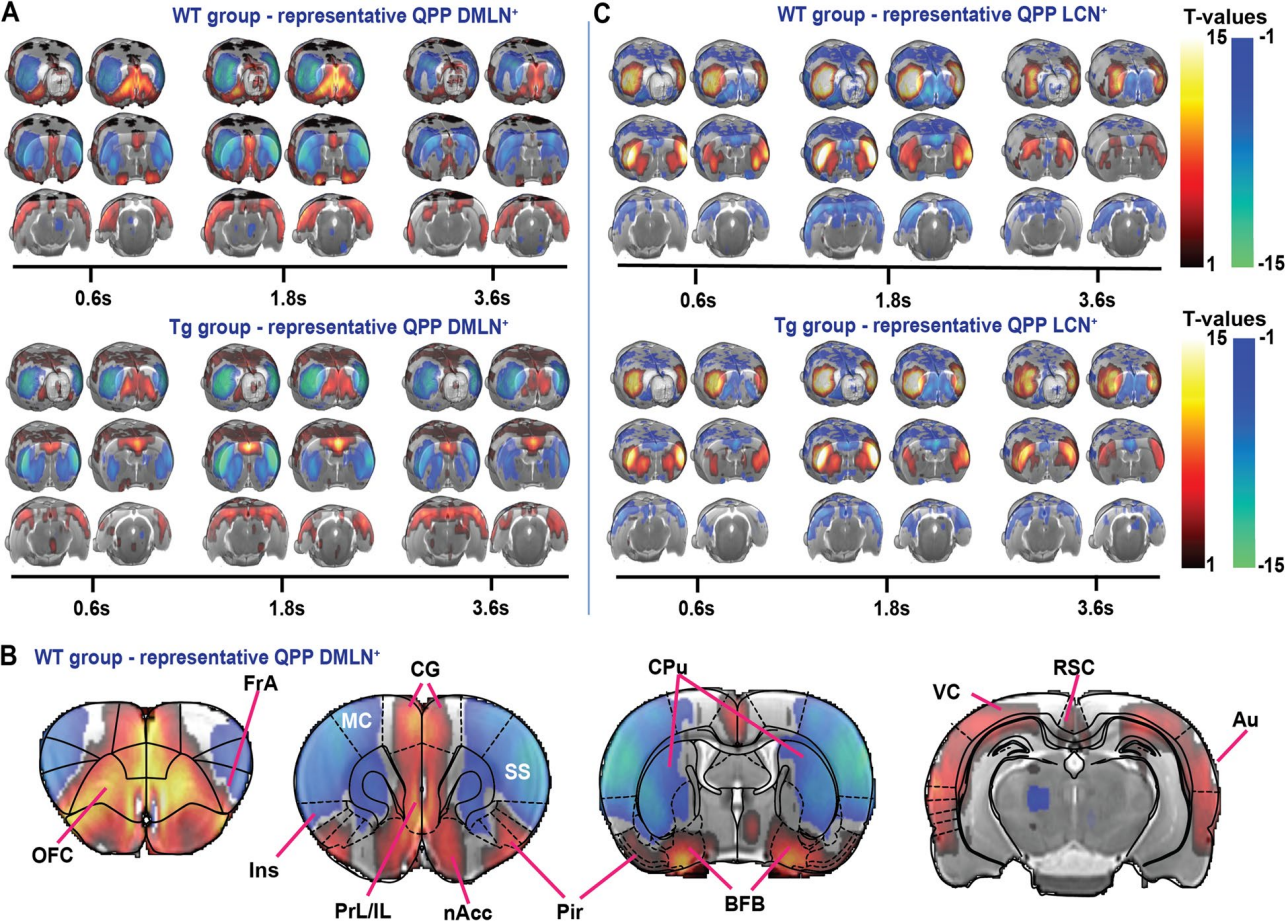
Visualization of group-level rQPPs of 4-month-old TgF344-AD rats (Tg) and wild-type littermates (WT). Results of the one-sample *t*-test (FDR *p* < 0.05, minimum cluster size 10 voxels) of QPP-DMLN (**A**) and QPP-LCN (**C**) visualized on the study-specific 3D template, demonstrating rQPPs for each experimental group. The T-maps represent statistically significant higher (red-yellow)/lower (blue-green) BOLD activity relative to the global mean BOLD signal (*p* < 0.05, FDR corrected). **B** Regional outlines of coronal slices of the Paxinos atlas overlaid on the T-map of QPP-DMLN^+^ of the WT. OFC orbitofrontal cortex, FrA frontal association cortex, MC motor cortices, SS somatosensory cortices, Cg cingulate cortex, PrL prelimbic cortex, IL infralimbic cortex, Ins insular cortex, nAcc nucleus accumbens, CPu caudate putamen, BFB basal forebrain, Pir piriform cortex, VC visual cortices, RSC retrosplenial cortex, Au auditory cortex

Subsequently, to potentially unravel subtle differences in network function that the static FC analysis was not sensitive enough to show, a quasi-periodic pattern (QPP) analysis was performed. Based on previous findings in mice, short 3.6-s QPPs were extracted within each group at 4 months of age. These short group-level QPPs were expected to reveal the brain dynamics of single brain states [29, 46]. Next, hierarchical clustering was performed on the 200 extracted QPPs to identify clusters of patterns representing unitary or very similar brain states based on the similarity between their spatial and temporal properties. Robust clusters were selected based on several criteria (see M&M 4.2.3). The clustering revealed two large clusters in the wild-type (WT) rats, whereas multiple smaller clusters could be observed in the TgF344-AD rats (Suppl. Figure 4). The QPPs with the highest occurrence rate were named as representative QPPs (rQPPs). Two anti-correlated group-level rQPPs were observed in the WT and TgF344-AD rats (Fig. 2). The first QPP, referred to as QPP-DMLN^+^, showed co-activation of regions belonging to the DMLN (i.e., CG, RSC, orbitofrontal cortex (OFC), prelimbic cortex (PrL), infralimic cortex, visual cortex, and auditory cortex) and co-deactivation of regions belonging to the lateral cortical network (LCN) (i.e., primary and secondary somatosensory cortices, primary and secondary motor cortices, frontal association cortex and the caudate putamen) (Fig. 2A, B). The second rQPP, referred to as QPP-LCN^+^, showed the opposite pattern with a deactivated DMLN and activated LCN (Fig. 2C). Both rQPPs showed a high spatial similarity between groups.

**Fig. 3:**
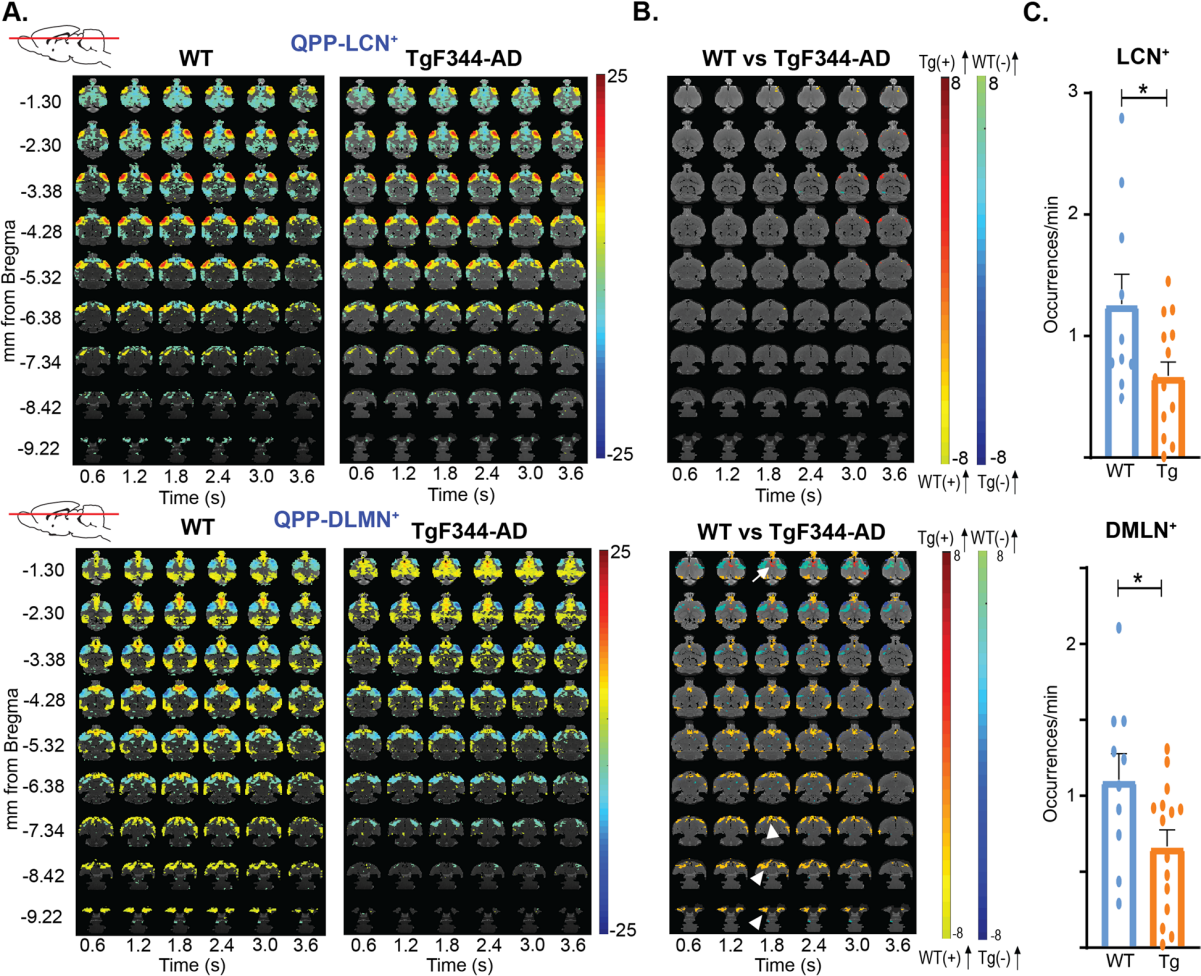
Spatiotemporal alterations in network activity in 4-month-old TgF344-AD rats (Tg) as compared to control littermates (WT). **A** T-maps (two-tailed one-sample *t*-tests, FDR *p* < 0.05, minimal cluster size 10) show the two rQPPs in each group. **B** T-maps of the two-sample *t*-test (two-tailed two-sample *t*-tests, FDR *p* < 0.05, minimal cluster size 10) between the rQPPs of TgF344-AD and WT rats. The warm colors (red/yellow) indicate a significant difference in BOLD activity between groups for voxels which are positive (activated) in the original QPP of the WT group. The right color bar (light blue/dark blue) indicates significant genotype differences for voxels which were deactivated in the original QPP of the WT group. For the QPP-DMLN, TgF344-AD rats demonstrate a significantly lower involvement of the OFC, PrL, Ent, and BFB is observed (arrowheads), together with a significantly increased activity of the Cg (arrow). For the QPP-LCN.^+^, only motor cortices and somatosensory cortex show significantly stronger activation in the TgF344-AD rats. **C** Comparison of cluster-wise occurrence rates between groups (mean ± SEM, two-sample *t*-test, two-tailed, FDR, *p* < 0.05) demonstrates significantly lower occurrences in the TgF344-AD rats for both QPPs. *p < 0.05

Alterations in neuronal activity can lead to differences in regional activation during certain brain states, which could induce spatial differences in activation during QPPs. Therefore, voxel-level co-activations and co-deactivations in both matched rQPPs were compared between groups (Fig. 3A, B). Significant differences in BOLD activity during QPP-LCN^+^ were observed and included higher involvement of the somatosensory and motor cortices in the TgF344-AD rats (Suppl. table 3). Moreover, differences in spatial BOLD activation were also observed during QPP-DMLN^+^ (Suppl. table 3). In 4-month-old TgF344-AD rats, BOLD activity was significantly reduced in regions belonging to the DMLN, mainly the entorhinal cortex, OFC, and PrL (Cohen’s *d* = 0.75). Interestingly, an increased BOLD activity was observed in the CG of TgF344-AD rats (Cohen’s *d* = 0.40), a hub region within the DMLN, indicating a potential disinhibition of this area in AD. In addition, the basal forebrain region (BFB) was found to be significantly co-activated with regions belonging to the DMLN in the WT animals, but not in TgF344-AD rats (Cohen’s *d* = 0.80). The observed loss of co-activation between the BFB and the DMLN in TgF344-AD rats during QPPs at the pre-plaque stage, as well as the disinhibition of CG that has strong connections to BFB, strongly corroborate the important role of BFB in the regulation of network activity and are consistent with the spatial alterations observed in QPP-DMLN^+^.

Having established alterations in the spatial structure of the QPP-DMLN^+^ in the TgF344-AD rats, we then proceeded to analyze if the frequency of occurrence of rQPPs would also change across the groups. Therefore, average occurrence rates were calculated for each group. Significantly decreased occurrence rates were observed for both the QPP-DMLN^+^ and the QPP-LCN^+^ clusters in the 4-month-old TgF344-AD rats when compared to WT (*p*_QPP-DMLN_ = 0.0427, *p*_QPP-LCN_ = 0.0449) (Fig. 3C) (for methods, see M&M 4.2.3), demonstrating that the instances of coordinated network activity were less common in the TgF344-AD animals during the pre-plaque stage.

**Fig. 4:**
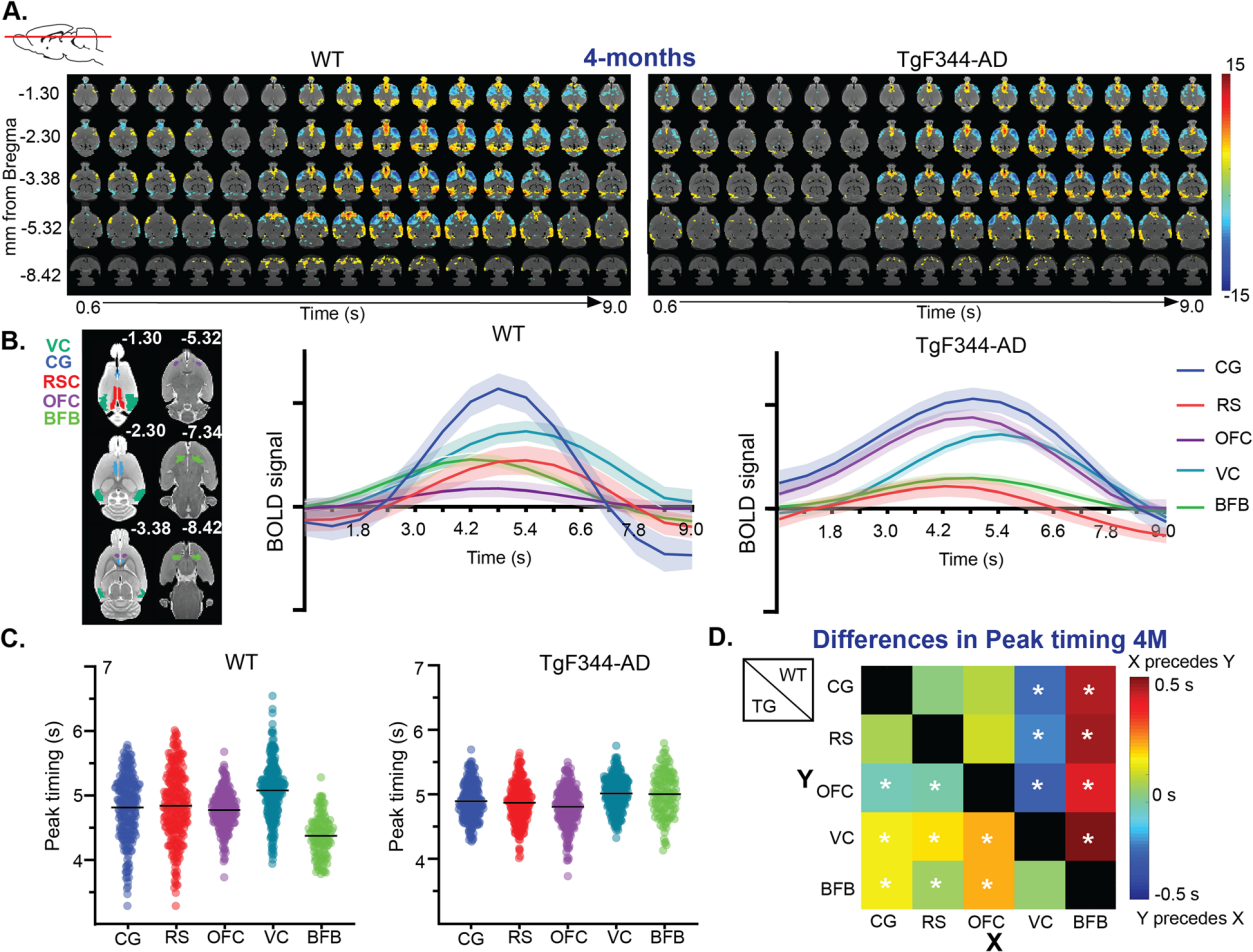
Long QPPs to investigate the propagation of network activity. **A** T-maps (two-tailed one-sample *t*-tests, FDR *p* < 0.05, minimal cluster size 10) show the long rQPP in each group. Colors indicate *T*-values. **B** Average BOLD time courses within regions of interest during 9-s QPPs. **C** Peak timings averaged across occurrences for each voxel within a certain region. Dots represent the distribution of average peak timing of individual region of interest (ROI)-specific voxels; the black lines represent the mean peak timing across all ROI-specific voxels. **D** Differences (peak_y_ − peak_x_) in mean peak timing across voxels for each connection. The top half represents the wild-type (WT) animals, while the lower half represents the TgF344-AD rats (Tg). Unpaired two-sample *t*-tests (FDR *p* < 0.05) between regions were performed to evaluate if peak timing was significantly different between ROIs. CG cingulate cortex, OFC orbitofrontal cortex, RSC retrosplenial cortex, HC hippocampus, BFB basal forebrain. *p < 0.05

As indicated above, short-duration QPPs resemble unitary brain states depicting co-modulation of brain regions around their peak activity. To investigate if other longer duration QPPs exist and/or to uncover the temporal evolution of brain activity around the short-duration QPPs, we proceeded with the detection of longer patterns (9 s = 15 TRs) in the two groups. In this case, spatiotemporal clustering resulted in 1 cluster of representative QPPs in each group (Suppl. Figure 5A). rQPPs were again visualized using a one-sample *t*-test, and QPPs were phase-aligned (Fig. 4A) (see methods 4.3.2). Visual inspection of the resulting pattern revealed that this 9-s-long rQPP showed the network activity prior to, and during the complete QPP-DMLN^+^ brain state. Thus, to better visualize the activity evolution around this brain state, we extracted the BOLD time courses of ROIs that demonstrated large differences in the short QPP-DMLN^+^ and averaged across rQPP occurrences (Fig. 4B). This revealed that peak activity in co-modulating ROIs showed differences in time. To statistically evaluate if activity in certain brain regions precedes the activity in other regions, differences in voxel-wise peak timing were investigated separately for the rQPP of each group. Propagation of BOLD activity across regions was evaluated by comparing the peak times of each voxel in a certain region of interest, averaged across different QPP occurrences. Statistical comparison of the distributions of voxel-wise peak times demonstrated that peak activity in the BFB, on average, preceded the peak activity in regions belonging to the DMLN in the WT animals, as is demonstrated by the significant difference in peak timings between the BFB and other regions within the WT animals (Fig. 4C, D, Suppl. Table 4). This indicates that the BFB was leading the DMLN activity during QPPs in the WT animals. In addition, peak activity within the visual cortex lagged behind peak activity in other regions of the DMLN. In the TgF344-AD rats on the other hand, BFB activity did not significantly precede the activity in other regions of the DMLN during QPPs. Instead, peak timing in the OFC preceded the peak timings in other regions, demonstrating an altered sequence of regional activation during QPPs in the TgF344-AD rats at the pre-plaque stage.

### Quasi-periodic pattern analysis in 6-month-old TgF344-AD rats and WT littermates

Since we observed large spatial and temporal differences in QPP activity during the pre-plaque stage, we wondered how these patterns of brain activity would develop at the early-plague stage given that scientists have previously shown that plagues at this AD stage have a neuroprotective role [47, 48]. To answer this question, rsfMRI and QPP analysis was performed on the same animals at 6 months of age when plagues start to appear in this animal model. Similar to the analysis at 4 months, two-hundred short-duration QPPs were extracted in each group and clustered using hierarchical clustering. In both groups, two clusters were identified, resulting in a DMLN-QPP^+^ and LCN-QPP^+^ (Suppl. Figure 6). Compared to the 4-month time point, 2-sample *t*-tests revealed less prominent inter-group differences in voxel-level co-activations in 6-month-old rats (effect size — Suppl. Table 3). Spatial alterations in QPP-LCN^+^ included a decreased BOLD activity in the somatosensory cortex, while the BOLD activity in the CPu was significantly higher in the TgF344-AD rats. When comparing cluster-averaged occurrence rates between groups, we observed no significant difference in occurrence rates for QPP-DMLN^+^ (*p* = 0.294847) and QPP-LCN^+^ (*p* = 0.126209). These results suggest that network activity during QPPs in TgF344-AD rats at pre-plaque stages normalized and closer resembled the activity of age-matched WT littermates.

The aforementioned results demonstrated the increased spatiotemporal similarity of short rQPPs between groups during the early-plaque stage. Given that the spatiotemporal changes coincided with altered sequential BOLD activity observed in TgF344-AD during the pre-plaque stage, we asked if at the early-plaque stage the sequence of activation was also closer to the WTs. Therefore, 9-s-long QPPs were extracted in each group. Spatiotemporal clustering again resulted in 1 cluster of robust QPPs in each group (Suppl. Figure 4). rQPPs were visualized using a one-sample *t*-test, and QPPs were phase-aligned and differences in voxel-wise peak timing were evaluated separately for the rQPP of each group (Suppl. Figure 7) (see methods 4.3.2). In WT rats, BFB activity preceded the activity in other regions of the DMLN, similar to what was observed at the pre-plaque stage in WT rats, which suggested BFB activity was leading the activity in the DMLN (Suppl. Figure 7C). In contrast, In TgF344-AD rats, peak activity in the OFC preceded the activity in other regions during the rQPP in the TgF344-AD rats, similar to the pre-plaque stage, which demonstrated that the altered sequence of activation was persistent at the early-plaque stage. To investigate if BFB peak timings differed between groups and across ages, an ANOVA analysis was performed on the voxel-wise peak timings in the BFB. Significant interaction effect (*p* = 0.0008), genotype effect (*p* ≤ 0.0001), and age effect (*p* = 0.0007) were observed. Post hoc Student *t*-tests (FDR *p* < 0.05) demonstrated a significant difference in the BFB peak timings between WT and TgF344-AD rats at both ages (*p*_4M_ ≤ 0.0001, *p*_6M_ ≤ 0.0001). In addition, a significant age effect was observed in the TgF344-AD rats (*p*_TgF344-AD_ ≤ 0.0001), but not in WT rats (*p*_WT_ = 0.9848), suggesting that, in contrast to the peak timing in the TgF344-AD rats, BFB peak timing in the WT remained stable over time.

### Immunohistochemical evaluation of neuroinflammation and synaptic alterations

**Fig. 5:**
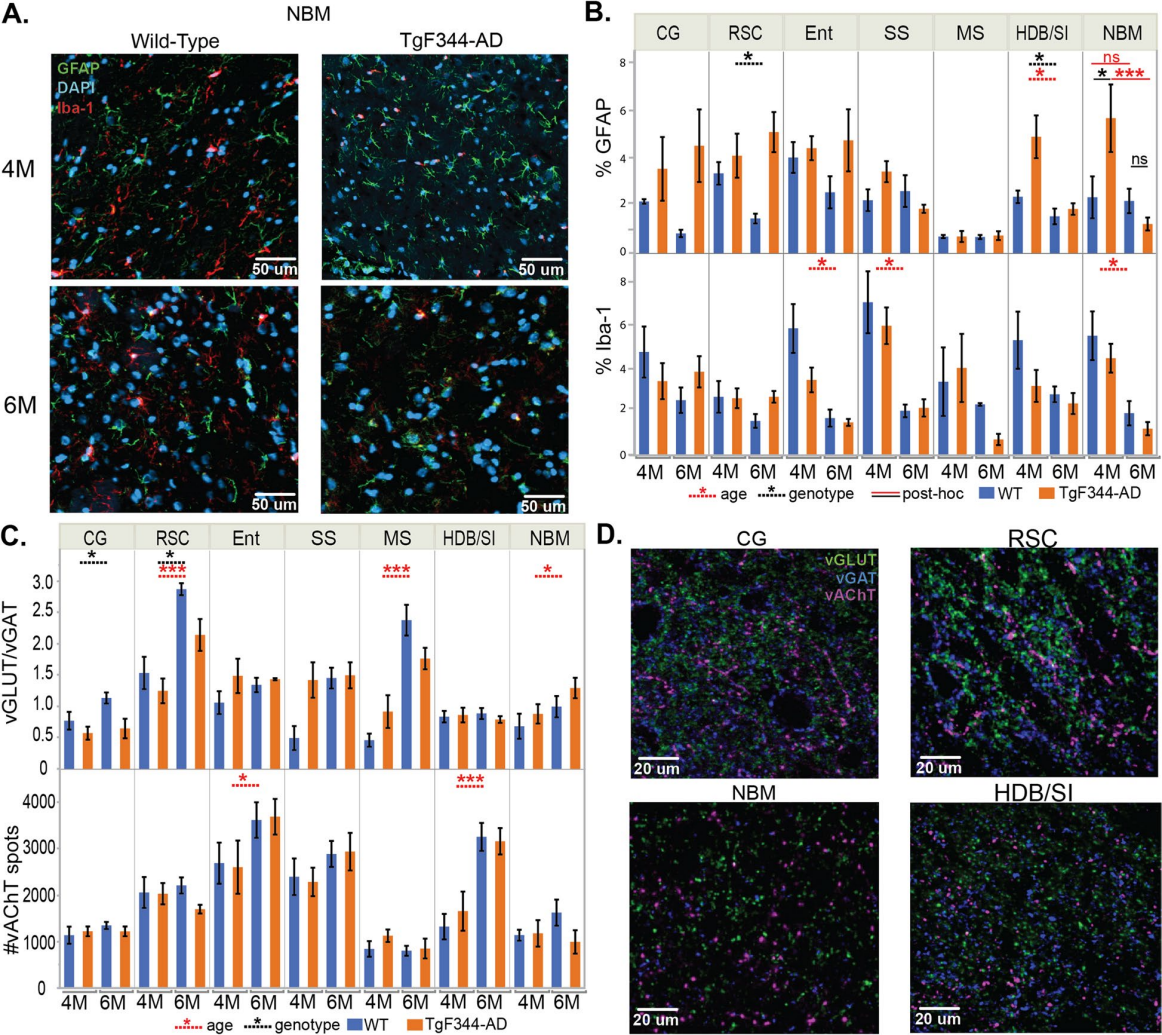
Histological evaluation of neuroinflammation and synaptic alterations in TgF344-AD rats. **A** Representative images showing astrocytes (green, GFAP) and microglia cells (red, Iba-1) in the nucleus basalis of Meynert (NBM). Nuclei were counterstained with DAPI (blue). Increased signal from GFAP (green) in the 4-month-old TgF344-AD rats. **B** Graphs represent the mean %area positive for GFAP (± SEM) (top) or %area positive for Iba-1 (± SEM) (bottom) for each brain region. Blue represents the wild-type (WT) animals, while orange represents the TgF344-rats. Dashed lines indicate significant genotype effects (black) or age effects (red). Solid lines indicate significant differences between genotypes (black) or ages (red) after the Student *t*-test (FDR *p* < 0.05) when genotype*age interaction was significant. **C** Graphs represent the mean vGLUT/vGAT ratio (top) and spot count of vAChT (bottom) (mean ± SEM). Blue represents the WT animals, while orange represents the TgF344-rats. Dashed lines indicate significant genotype effects (black) or age effects (red). **D** Representative images of the triple staining (vGLUT in green, vGAT in blue, vAChT in magenta) of three different regions in a representative 4-month-old WT rat. *p < 0.05, **p < 0.01, ***p < 0.001. CG cingulate cortex, RSC retrosplenial cortex, Ent entorhinal cortex, SS somatosensory cortex, MS medial septum, HDB/SI horizontal limb of the diagonal band of Broca/substantia innominata, NBM nucleus basalis of Meynert, vAChT vesicular acetylcholine transporter, vGAT vesicular GABA transporter, vGlut vesicular glutamate transporter

Accumulation of (soluble) Aβ and pTau is associated with an increased abundance of (reactive) astrocytes and microglial cells. To investigate if neuroinflammation was present in the TgF344-AD rats during the pre- and early-plaque stages, immunohistochemistry was performed using glial fibrillary acidic protein (GFAP) and Iba-1 staining for astrocytes and microglial cells respectively (Fig. 5 A, B) in 7 different brain regions, which either showed differences in the QPPs (CG, RSC, Ent, SS) or regions which are part of the basal forebrain nuclei (MS, HDB/SI, NBM). Statistical analysis (two-way ANOVA, age, genotype, age*genotype) revealed a significant interaction between age and genotype in the nucleus basalis of Meynert (NBM) (*p* = 0.0262) (Suppl. Table 5). Post hoc analysis demonstrated a significantly higher GFAP signal in the NBM in 4-month-old TgF344-AD rats compared with age-matched WT (*p* = 0.0366), which was not present at 6 months (*p* = 0.5880). Moreover, a significant decrease in GFAP levels over time was observed in tissue slices from TgF344-AD rats (*p* = 0.0072), an effect which was not present in the WT (*p* = 0.9064). Interestingly, a significant genotype effect (*p* = 0.0269) was observed in another nucleus of the basal forebrain, the horizontal limb of the diagonal band of Broca and substantia innominata (HDB/SI), demonstrating an increased abundance of astrocytes in the TgF344-AD rats, which was mainly present at 4 months of age. These results suggest that the astrogliosis in the NBM and HDB/SI is present during the pre-plaque stage, but disappears at the early-plaque stage in TgF344-AD rats. Moreover, significantly higher levels of GFAP were observed in the RSC of TgF344-AD rats (genotype *p* = 0.0372), mainly at 6 months of age. In the RSC, increased astrogliosis is observed at both ages. When comparing the microglial abundance, no significant genotype differences were observed. However, a significant age effect was observed in the MS, Ent, NBM, and SS (Suppl. Table 6), indicating a decrease of microglial abundance over time.

Increased concentrations of (soluble) Aβ and pTau might induce alterations in neurotransmission of GABA-ergic and glutamatergic neurons, which could result in an imbalance between excitatory and inhibitory synapses. Studies have demonstrated that alterations in the excitatory/inhibitory balance could induce alterations in BOLD activity. To investigate if alterations in cortical and subcortical excitatory/inhibitory (E/I) balance were present in the TgF344-AD rats in regions which demonstrated altered regional activation during QPPs, a histological analysis of glutamatergic and GABA-ergic synapses was performed. The ratio of the number of glutamatergic and GABA-ergic synapses (vGlut/vGAT) was compared between groups using a two-way ANOVA (Fig. 5C). The analysis demonstrated a significant genotype effect in the RSC and CG, indicating a shift in E/I balance towards decreased excitation or increased inhibition in the TgF344-AD rats. No significant genotype effects were observed in other brain regions. Significant age effects were observed in the RSC, MS, and NBM of the WT and TgF344-AD rats indicating an increased vGlut/vGAT ratio (Suppl. Table 7).

Cholinergic neurons which reside in the BFB and project to the entire cortex have been implicated in the modulation of cortical networks and are known to be vulnerable for (soluble) Aβ-induced toxicity. We hypothesized that alterations in cholinergic signaling could be responsible for the spatiotemporal alterations observed in the QPPs during the pre-plaque stage. Therefore, immunohistochemical evaluation of cholinergic synapses was performed to evaluate if the abundance of cholinergic synapses was decreased in the cortex and BFB. Synaptic counts for cholinergic synapses (vAChT) demonstrated significant age effects in the Ent and HDB/SI, where the cholinergic synaptic density at 6 months was increased compared to 4 months in both groups. However, no significant genotype effects were observed in the abundance of cholinergic synapses (Fig. 5D, Suppl. Table 8).

## Discussion

This study investigated whole-brain, recurrent patterns of brain activity, at early stages of AD in a highly translational rat model of AD. At 4 months of age, during the pre-plaque stage, significant inter-genotype differences in voxel-level activation were observed in QPP-DMLN^+^, which mainly involved decreased BOLD activity in the BFB and regions belonging to the DMLN, together with an increased BOLD activity in the CG. Moreover, peak BOLD activity in the BFB preceded peak activity in other regions in wild-types, but not in TgF344-AD rats. These results suggest differences in neuronal mechanisms modulate the spatial involvement of brain regions in TgF344-AD rats. The differences in network activity coincided with astrogliosis, limited to the NBM, HDB/SI, and RSC in the absence of Aβ depositions and pTau in cortical and subcortical regions. In addition, a decreased E/I ratio was observed at the pre-plaque stage, suggestive of increased inhibition or decreased excitation in the CG and RSC, two hub regions of the DMLN. However, at 6 months of age, during the early-plaque stage, spatiotemporal properties of QPPs in the TgF344-AD rats were more similar to the WT littermates, while an altered sequence of BOLD activation persisted in the TgF344-AD rats. Interestingly, astrogliosis in the BFB was absent at the early-plaque stage, while decreased excitation and/or increased inhibition in the CG and RSC persisted at the early-plaque stage, suggesting an important role of early astrogliosis in the NBM in global network alterations.

### Static functional connectivity vs QPP analysis of rsfMRI data in TgF344-AD rats

Previous research involving QPP analysis investigated the relationship between QPPs and static FC, by evaluating FC before and after regression of QPPs in humans. They observed a decrease in sFC between the default mode network and task-positive network after the regression of the main QPP, demonstrating that QPPs contribute to static FC [27]. Thus, the synchronous co-activation events that QPPs reflect do contribute to static FC. However, sFC is calculated based on the whole BOLD time course, on which the short window QPPs, demonstrating temporary high FC, only have a small contribution. The current study performed a region-of-interest-based analysis of static FC which did not result in significant differences between genotypes after multiple comparison correction. On the other hand, we did observe significant spatial alterations in QPPs in several regions, mainly belonging to the DMLN. When looking at the observed differences in sFC, without FDR correction (Suppl. Figure 3, Suppl. Table 2), differences between regions belonging to the LCN and DMLN were observed at 4 months of age, consistent with our QPP analysis. In addition, sFC differences between the BFB and DMLN regions were observed at the pre-plaque stage. These differences did not survive the FDR correction, but it does indicate that the changes observed in the QPPs are to a lesser extent present in the sFC differences at 4 months of age. In addition, at 6 months, differences in sFC become less abundant, similar to what we observe in the QPPs at that time point. These results suggest that QPP analysis is more sensitive to early alterations in network function than static FC analysis.

### AD pathology and behavioral alterations in TgF344-AD rats

Amyloid pathology is one of the major histopathological hallmarks of AD. Cortical and hippocampal Aβ plaques have been observed from 6 months onwards in TgF344-AD rats, as is observed in this study [35, 49]. At 4 months of age, Aβ plaques were absent, as has been observed before in 4-month-old TgF344-AD rats [49]. The current study observed increased concentrations of soluble Aβ species in 4-month-old TgF344-AD rats, which is in line with the results of a recent study which observed increased concentrations of soluble Aβ1-40 and Aβ1-42 in blood samples of 3-month-old TgF344-AD rats [50]. A previous study has observed pTau accumulation in the locus coeruleus observed at 6 months of age in TgF344-AD rats [37], similar to our observation in the current study. However, no other studies have investigated pTau accumulation in other brain regions at 4 and/or 6 months of age. Only a few studies have investigated cognitive and behavioral alterations in 4-month-old TgF344-AD rats. The two studies from two different facilities have observed altered spatial navigation and spatial memory in 4-month-old TgF344-AD rats using frequently used behavioral tests (e.g., Barnes Maze and Active Allothetic Place Avoidance test) [51, 52]. Another research group observed impairments in working memory in 5-month-old TgF344-AD rats, but not at later time points [53]. In contrast, a different research group did not observe significant differences in spatial memory in 4-month-old TgF344-AD rats in the Morris water maze, but did find differences at later time points [54]. These results clearly illustrate the variability of the behavioral test results, which are possibly induced by the use of different behavioral assays and different research groups. Nevertheless, the current study observed altered activity in the BFB, of which the cholinergic projections to the hippocampus, RSC, and parietal cortex are important in spatial navigation and spatial memory [55]. The altered activity in the BFB and DMLN observed in the QPPs could be indicative of cognitive impairments. Moreover, the current study observed astrogliosis in the NBM and RSC, together with a decreased E/I ratio in the RSC, which might be important cellular pathological processes contributing to the behavioral alterations observed in aforementioned studies.

### Spatial and temporal alterations in DMLN activity in AD

The observed QPPs in this study demonstrated two unitary brain states, namely an activated state of DMLN with LCN deactivated (i.e., QPP-DMLN^+^) and an activated state of LCN with DMLN deactivated (i.e., QPP-LCN^+^). Similar QPPs have been observed in mice and humans (DMN and TPN) [29, 56, 57]. In TgF344-AD rats, we found a decreased frequency of occurrence of both short-duration QPPs and the presence of prominent spatial alterations in QPP-DMLN^+^ during the pre-plaque stage. Key differences consisted mainly of a decreased co-activation of the BFB with the DMLN, a decreased BOLD activity within the DMLN, and an increased BOLD activity in the CG in TgF344-AD rats. These results are consistent with similar observations describing changes in the QPP-DMN^+^ in a recent study in patients with subjective cognitive decline, a self-perceived cognitive deterioration which is thought to be associated with an increased risk for developing AD-related MCI. Using dynamic FC analyses in healthy controls and patients with subjective cognitive decline, Liang et al. observed decreased occurrence of a brain state where DMN was activated, while other networks were deactivated [58]. This brain state resembles the QPP-DMLN observed in this study. The analogy between the findings of Liang et al. and the current study suggests similar alterations in DMLN activity are observed in patients with subjective cognitive decline and at pre-plaque stages in the TgF344-AD rat.

### BFB dysfunction in AD

Changes in BFB volume measured by MRI have been observed at early stages of AD in patients and have been proven to be related to early BFB degeneration [15, 59, 60]. Moreover, decreases in BFB volume are predictive of an increased risk of conversion from mild cognitive impairment (MCI) to dementia [61]. During the pre-symptomatic, prodromal stages, BFB degeneration, detected with volumetric MRI analysis, precedes the spread of cortical Aβ pathology [62, 63]. Since functional changes precede volumetric changes, BFB FC could be a potential earlier biomarker for AD. A recent study in patients with subjective cognitive decline demonstrated a significantly decreased FC between the BFB and the hippocampus [64]. In addition, a recent study observed significant correlations between BFB connectivity, cerebrospinal fluid biomarkers and blood serum biomarkers of AD in patients at different stages of the AD [65], strengthening the hypothesis that alterations in BFB FC could be used to detect AD at early stages. Future experiments using QPP analysis on data of (pre-symptomatic) AD patients would be valuable to investigate if our finding can be translated to human AD, i.e., study if the disconnection between the BFB and the DMN during QPPs could be used as an early biomarker of AD.

### Altered BFB modulatory function during whole-brain network activity in AD

The mechanisms leading and modulating the whole-brain network activity are still poorly understood. A recent study in humans investigated the propagation of activity during QPPs across different brain regions, in order to elucidate brain regions driving the QPP activity. In all QPPs observed in humans, peak activity within the thalamus, brainstem, and deep nuclei preceded cortical activity [57]. Partially in agreement with the findings in humans, the current study shows that in WT rats at both ages, the peak of BOLD activity within the BFB precedes the activity within the DMLN, suggesting an important role of the BFB in coordinating the network activity in rats and humans. Further evidence of the modulating role of the BFB in human network activity was presented in a recent study by Harrison et al. [66], where they observed that the deactivation of the BFB precedes the deactivation of the DMN during tasks in human subjects. Moreover, dynamic causal modeling, an analysis technique used to assess the direction of FC, confirmed that the BFB was driving the changes in DMN upon task initiation [66]. In addition, other studies state that projections from the BFB are thought to drive waves of propagating activity from anterior to posterior brain areas, as is observed in the QPPs [67, 68]. Together, these observations support our hypothesis that the BFB could act as an important leader and modulator of whole-brain network activity during QPPs in healthy humans and rats. In the TgF344-AD rats, differences in propagation of activity within the DMLN were observed at both ages, where activity in the BFB does not precede the activity in the DMLN. Instead, the activity in the OFC precedes the activity in other regions. These findings imply that different neuronal mechanisms could be leading the QPPs in the TgF344-AD rats, in the absence of the modulating input from the BFB. Further research focusing on causal connections between the BFB and DMLN regions could provide valuable insights into the direction of information flow in TgF344-AD rats at different disease stages, which might be translated to early stages of human AD.

### Alteration in excitatory/inhibitory balance in AD

The rsfMRI results of the current study demonstrate an increased similarity of spatiotemporal properties of QPPs between WT and TgF344-AD rats at the early-plaque stage, while at the pre-plaque stage, large spatiotemporal differences are observed (Suppl. Table 3). This could imply that compensatory mechanisms are at play in the early-plaque stage that counteract the alterations in network activity observed during the pre-plaque stage. In line with the observations of the current study, compensatory mechanisms occurring at early stages of AD have been observed in different mouse models of AD [69, 70]. Previous work from our lab demonstrated an imbalance in excitatory and inhibitory transmission coincided with alterations in FC in the DMLN in an amyloidosis mouse model of AD. Moreover, paradoxical upregulation of presynaptic glutamatergic synapses has been observed in the frontal cortex of MCI patients [71], in the absence of alterations in GABA-ergic synapses [71, 72] (for a review [73]). In addition, a recent study demonstrated increased activity of interneurons at the pre-plaque stage in APP/PS1 mice [74]. In the current study, the excitation-inhibition balance was evaluated within each region in terms of the ratio of synaptic density of glutamatergic and GABA-ergic markers. We demonstrate that this ratio was decreased in the RSC and CG both at the pre-plaque and early-plaque stage in the TgF344-AD rats, indicating a shift towards decreased excitation or increased inhibition in these cortical regions. This is in concordance with the aforementioned hyperexcitability of interneurons in APP/PS1 mice [74]. However, the current results are not in concordance with the aforementioned observations of increased glutamatergic synaptic density at early stages of AD in MCI patients. This might be explained by the fact that the pre-plaque and early-plaque stages in the TgF344-AD rat resemble early preclinical stages, not MCI stages in patients [35, 75]. Histological evaluation of synaptic markers in aged TgF344-AD rats, resembling the MCI stage, might reveal similar changes in synaptic density as is observed in MCI patients. Moreover, the apparent restoration of WT-like QPP patterns in the TgF344-AD rats at the early-plaque stage could not be accounted for using histological results at the same stage.

### Cholinergic synaptic dysfunction in AD

The cholinergic neurons in the BFB are highly susceptible to early AD pathology, hence the observation that the BFB is one of the first regions affected in AD. Ex vivo studies in patients with limited Aβ pathology, observed increased metabolic activity and increased expression of genes involved with synaptic activity in cholinergic neurons in the NBM [76–78]. Moreover, the density of cholinergic synapses in the cortex was increased, during the pre-plaque stage in a mouse model of AD, followed by decreased cholinergic density at advanced stages of AD [79]. The current study did not observe significant differences in the number of cholinergic synapses in the cortex or the basal forebrain regions in the TgF344-AD rats at the pre-plaque, nor at the early-plaque stage. This is in agreement with a post-mortem study in AD patients that only observed loss of cholinergic synapses during the late symptomatic stage [80], but not with aforementioned studies which observed increased cholinergic synaptic density and increased cholinergic activity. This might be explained by the differences between animal models and differences in sensitivity of the detection method. Moreover, the absence of alterations in cholinergic synaptic density does not exclude alterations in cholinergic synaptic transmission at the molecular level, since soluble Aẞ monomers and oligomers have been shown to interfere with cholinergic synaptic signaling (for a review, [81]). However, future research regarding cholinergic neurotransmission in early TgF344-AD rats is needed to validate this hypothesis. A recent study demonstrated an age-dependent increase in nicotinic acetylcholine receptors in WT F344 rats starting from 6 months onwards, which was absent in age-matched TgF344-AD rats, leading to a significant difference in receptor density between genotypes at 9 months of age [82]. Similarly, the current study observed age effects in the HDB/SI and Ent demonstrating an increased cholinergic synaptic density in both groups at 6 months of age, without significant differences between groups.

### Astrogliosis might contribute to spatiotemporal alterations in activity patterns during the pre-plaque stage

Histological analysis in 4-month-old TgF344-AD rats demonstrates astrogliosis in the NBM and HDB/SI, which diminishes at 6 months of age. Similar trends have been observed in a mouse model of AD, where astrogliosis was observed during the pre-plaque stage in APP/PS1 mice, which decreased at the early-plaque stage [83]. Interestingly, the astrogliosis during the pre-plaque stage which diminishes at the early-plaque stage is comparable to the observations in the rsfMRI data, where the spatiotemporal properties of the QPPs in the TgF344-AD rats are also more similar to the WT littermates at 6 months of age. Astrocytes are important in synaptic homeostasis and regulation of glutamatergic neurotransmission by the reuptake of glutamate from the synaptic cleft [84–88]. Moreover, reactive astrocytes in AD induce local changes in GABA-ergic and cholinergic signaling [89]. Animal studies have demonstrated that modulation of specific neuronal populations within the basal forebrain, including GABA-ergic and cholinergic neurons, induces alterations in BOLD fluctuations and electrical activity within the DMLN in macaques and rats [4, 9, 13, 90]. Given the fact that projections from the BFB to the cortex are important modulators of BOLD activity, alterations in local neurotransmission in the NBM and HDB/SI, due to astrogliosis, might contribute to the spatial alterations in whole-brain patterns of activity observed in the current study at the pre-plaque stage. Interestingly, a recent study published in bioRxiv observed that early FC disruptions in the CG, which was observed in APP^NL−F^ mice before plaques were present in the brain, were accompanied by decreased astrocytic signaling. Moreover, Shah et al. demonstrated that astrocytes have an important regulatory function of network activity, which was affected in early AD [91]. This is in agreement with the findings of the current study where astrogliosis in the BFB is coinciding with large spatiotemporal differences in BOLD activity in BFB. Further research focusing on the role of astrogliosis in network alterations at the pre-plaque stage of AD is necessary to validate this hypothesis and could offer valuable insights into novel therapeutic strategies to counteract network imbalance at preclinical stages of AD.

### Limitations of the study

There are several limitations to the study. First, the rsfMRI scans in this study were performed in isoflurane- and medetomidine-anesthetized rats. This combination is known to reduce FC in the subcortical structures such as the hippocampus, a structure affected early in AD. Alterations in network activity in the hippocampus could not be evaluated in the current study. However, a rsfMRI study has demonstrated that FC under this anesthesia protocol is very close to the FC patterns observed in awake rats [92]. In addition, it would be valuable to investigate early network dysfunction not at rest, but during a task. Functional MRI studies at pre-plaque and early-plaque stages of AD could offer novel insights into network dysfunction of task-related networks. Second, this study only used male rats to limit the variability and the number of animals used in this study, since previous FC studies observed differences in static FC between genders. However, studies have demonstrated that disease progression is faster in women compared to men and sex differences have also been observed in animal models for AD [93]. A recent study in TgF344-AD rats showed that amyloid pathology and neuroinflammation are more severe in females. But, cognitive and behavioral deficits were found to be less severe in female rats compared to male rats [94]. Moreover, an electrophysiology study demonstrated that the onset of synaptic dysfunction in the hippocampal circuit occurs later in females (9 M) compared to males (6 M), possibly due to the neuroprotective effects of estrogen [95], demonstrating that gender differences are also present in TgF344-AD rats. Future studies including females would be valuable to validate the current findings in female TgF344-AD rats and to improve translation to humans. Third, the current study did not investigate the pathological mechanisms underlying the astrogliosis in the BFB in the absence of amyloid plaques. Future experiments focusing on the mechanisms underlying the increased astrocyte abundance and the role of inflammation in the progression of AD would be valuable to evaluate if this astrogliosis is a valuable therapeutic target. In addition, the histological analysis was not performed on the same animal cohort as the rsfMRI, limiting the possibility to correlate the histological findings to the MRI results. Future studies correlating MRI results to histological alterations should be performed to validate our findings. The biochemical analysis was performed on brain homogenate of intact hemispheres. It would be valuable to perform amyloid ELISA’s on specific brain regions, to gain a better understanding of which regions are affected first by soluble Aβ species. The immunohistochemical stainings of the GFAP and Iba-1 of the 4-month-old animals and the 6-month-old animals were performed in two separate batches, which could influence the quality of the stainings, inducing false positive age effects. In addition, Iba-1 and GFAP are general markers for astrocytes and microglial cells and therefore not only stained activated astrocytes and microglial cells. Future stainings to evaluate neuroinflammation would benefit from using markers which exclusively target activated microglia and astrocytes. Moreover, the study did not include any behavioral evaluation of memory function. It would be interesting to include a memory task to investigate how the altered network activity correlates to alterations in behavior at pre-plaque and early-plaque stages of AD. To conclude, the current study only focused on early stages of AD, which are thought to correspond to preclinical stages in humans. Including a later time point corresponding to MCI patients would be valuable to evaluate how network activity changes during healthy aging and AD progression.

## Conclusions

In conclusion, our data demonstrates that QPP analysis on rsfMRI data is a promising tool to evaluate spatiotemporal alterations in brain activity during early stages of AD. The results provide insights into macroscale changes in network function in AD at pre-plaque and early-plaque stages, which are thought to be caused by altered BFB function. This observed altered BFB activity during the QPPs might prove to be an important biomarker of pre-symptomatic AD, possibly aiding early detection and early intervention in AD. Future research using QPP analysis in (pre-symptomatic) AD patients would be valuable to examine the translational potential of the current findings. Moreover, further research of the particular role of astrogliosis in the basal forebrain nuclei remains necessary to gain a better understanding in order to elucidate potential therapeutic targets to restore the aberrant network function at preclinical stages of AD. 

## Supplementary Information

**Additional file 1:** Supplementary Information.

## Data Availability

The complete MRI dataset generated during this study and the complete histological and the Matlab scripts for the ROI-based analysis and QPP analysis of the rsfMRI data are available from the corresponding authors on reasonable request. In addition, the QuPath scripts for the analysis of the pTau, GFAP, and Iba-1 stainings are available from the corresponding authors on request. The codes for the analysis of the synaptic counts are openly available on https://github.com/DeVosLab/SynapseDetection.

## Abbreviations

AD: Alzheimer’s disease
Aβ: Amyloid-beta
pTau: Hyperphosphorylated tau
BFB: Basal forebrain
RsfMRI: Resting state functional magnetic resonance imaging
FC: Functional connectivity
BOLD: Blood oxygen level dependent
DMLN: Default mode-like network
QPP: Quasi-periodic pattern
LCN: Lateral cortical network
CG: Cingulate cortex
RSC: Retrosplenial cortex
NBM: Nucleus basalis of Meynert
LC: Locus coeruleus
Ent: Entorhinal cortex
HDB: Horizontal limb of the diagonal band of Broca
SI: Substantia innominata
MS: Medial septum
DG: Dentate gyrus
Hipp: Hippocampal network
Sens: Sensory cortical network
SS: Somatosensory cortex
BFB: Basal forebrain
OFC: Orbitofrontal cortex
PrL: Prelimbic cortex
ROI: Region of interest
CPu: Caudate putamen
GFAP: Glial fibrillary acidic protein
vAChT: Vesicular acetylcholine transporter
vGAT: Vesicular GABA transporter
vGlut: Vesicular glutamate transporter
