# Supplementary material for: Altered basal forebrain function during whole-brain network activity at pre- and early-plaque stages of Alzheimer’s disease in TgF344-AD rats
Source: Alzheimers Res Ther. 2022 Oct 10;14:148. doi: 10.1186/s13195-022-01089-2 (PMC9549630; doi:10.1186/s13195-022-01089-2)
Supplement: Supplementary file 1 — Additional file 1: Supplementary Information. [file 13195_2022_1089_MOESM1_ESM.docx]

# Supplementary figures

**
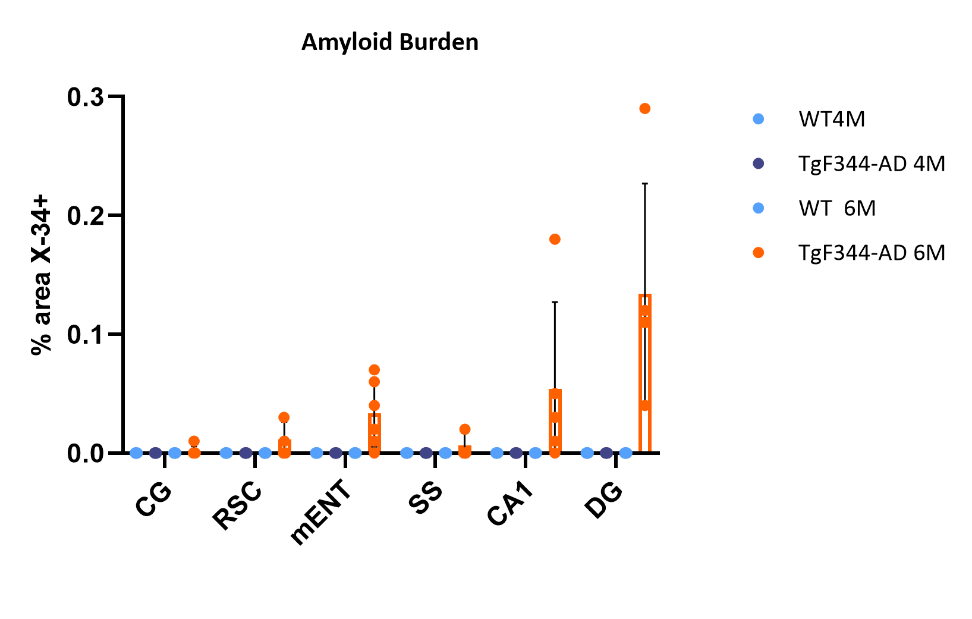
**

**Supplementary figure 1: Amyloid burden in TgF344-AD rats.** Bar graphs represent the mean (+/- SEM) %area positive for amyloid plaques (X-34).


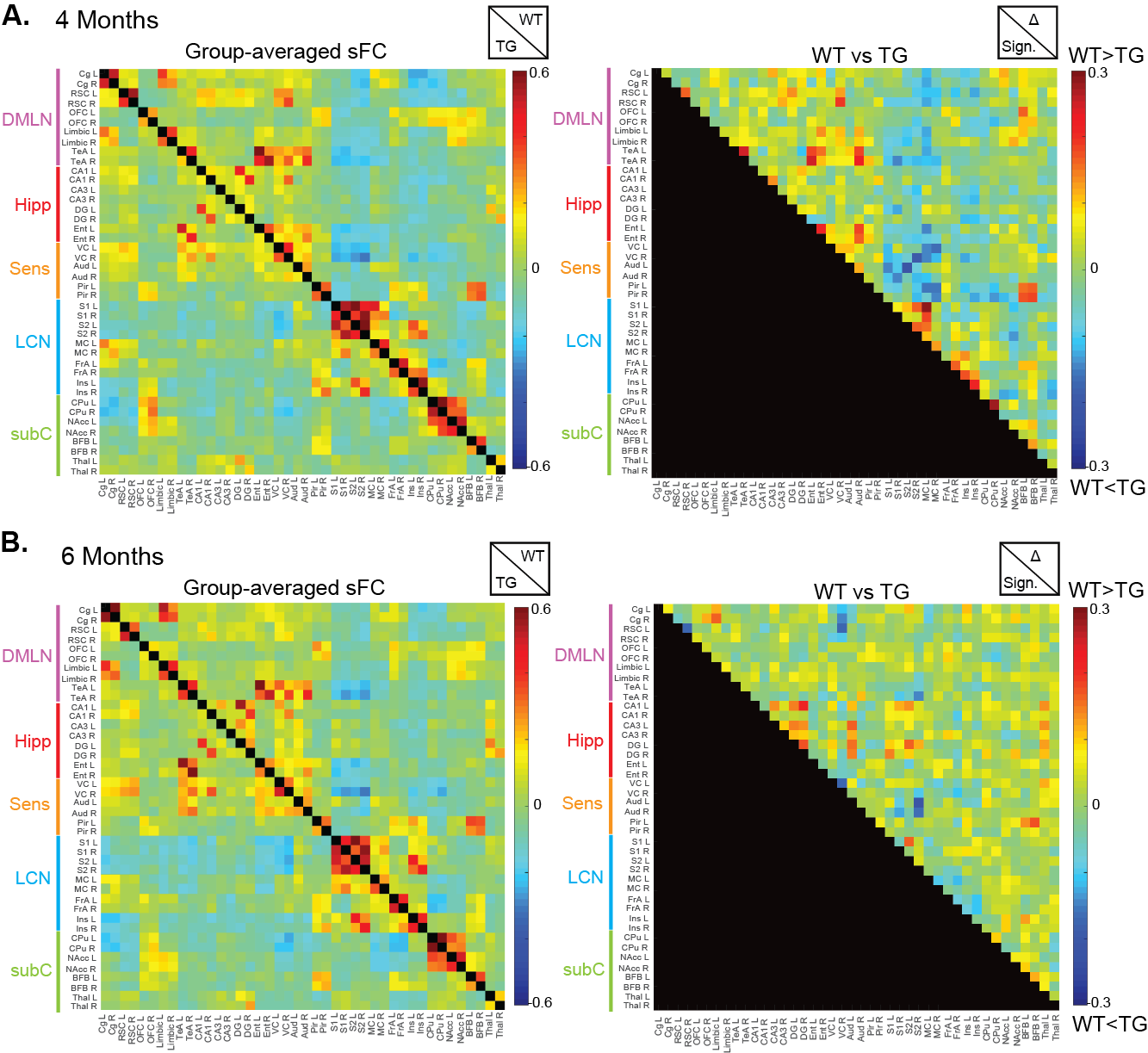


**Supplementary figure 2: ROI based analysis demonstrates no significant differences in static FC between TgF344-AD (TG) rats and Wild-type (WT) littermates.** ROI based matrices which displays the z-scored FC between ROI-pairs for the WT (top half of matrix) and TG (bottom half of matrix) at 4 months of age (A) and 6 months of age (B). Two-sample t-test (FDR p<0.05) demonstrates no significant differences in FC between groups, as is shown on the right matrix. DMLN = default mode-like network, Hipp = hippocampal network, Sens = sensory network, LCN = lateral cortical network, subC = subcortical network, L = left, R = right, Cg = cingulate cortex, RSC = retrosplenial cortex, OFC = orbitofrontal cortex, TeA = temporal association cortex, DG = dentate gyrus, VC = visual cortex, Aud = auditory cortex, Pir = Piriform cortex, S1 = primary somatosensory cortex, S2 = secondary somatosensory cortex, MC = motor cortex, FrA = frontal association cortex, Ins = insular cortex, CPu = caudate putamen, NAcc = nucleus Accumbens, BFB = basal forebrain, Thal = thalamus

**
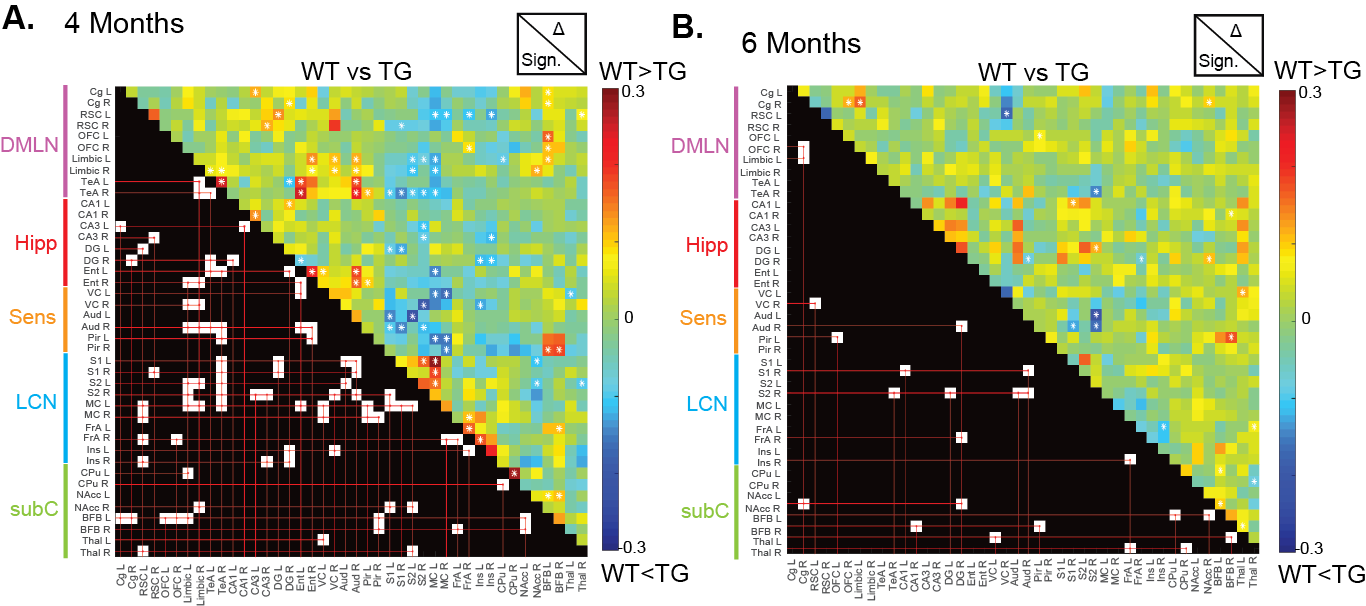
Supplementary figure 3: ROI based analysis without FDR correction.** FC difference matrices which displaying the results of an uncorrected two-sample t-test demonstrating significant differences in FC (white boxes lower half of the matrix) which do not survive FDR correction. DMLN = default mode-like network, Hipp = hippocampal network, Sens = sensory network, LCN = lateral cortical network, subC = subcortical network, L = left, R = right, Cg = cingulate cortex, RSC = retrosplenial cortex, OFC = orbitofrontal cortex, TeA = temporal association cortex, DG = dentate gyrus, VC = visual cortex, Aud = auditory cortex, Pir = Piriform cortex, S1 = primary somatosensory cortex, S2 = secondary somatosensory cortex, MC = motor cortex, FrA = frontal association cortex, Ins = insular cortex, CPu = caudate putamen, NAcc = nucleus Accumbens, BFB = basal forebrain, Thal = thalamus


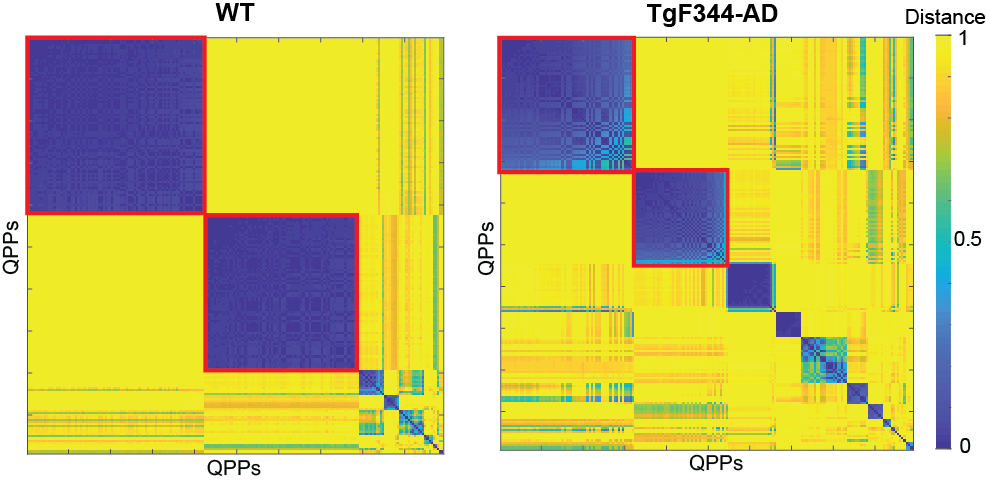


**Supplementary figure 4: Hierarchical clustering based on Spatiotemporal properties of QPPs of 4-month-old WT and TgF344-AD rats.** Distance matrices of the clustering solution in the wild-type (WT) animals (left) and TgF344-AD rats (right). Colors indicate the distance between a pair of QPPs. Red squares mark the clusters of QPPs which were robust according to the criteria, and which were used in further analysis.


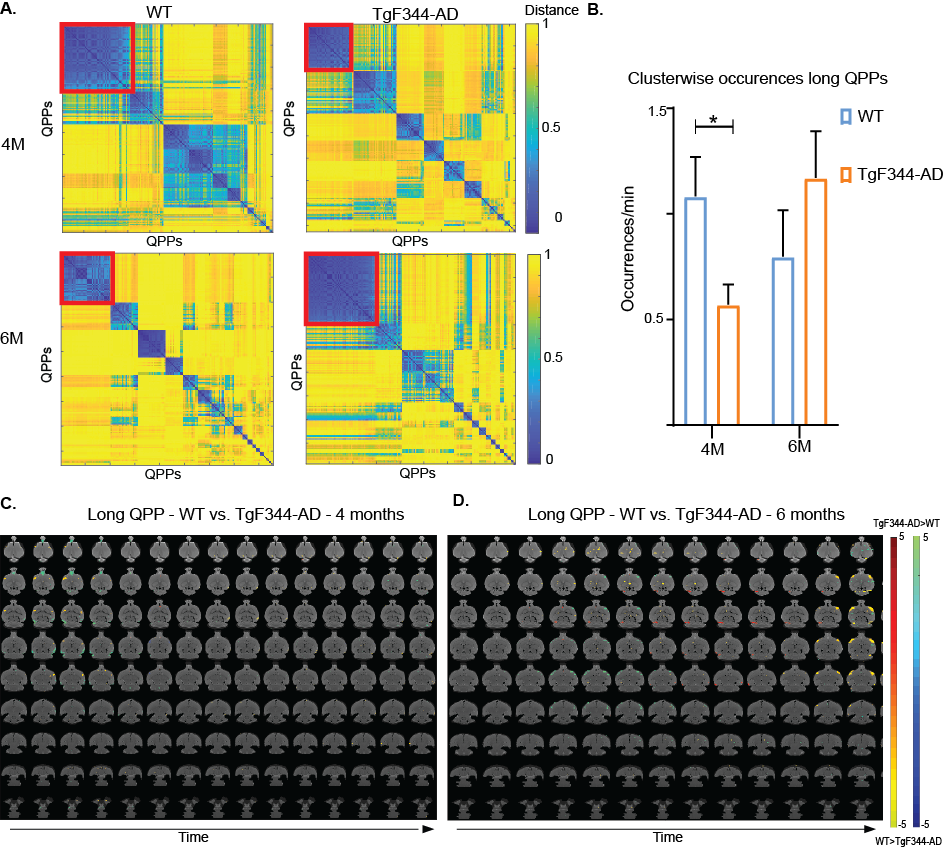


**Supplementary figure 5: Analysis of 9 second long QPPs at 4 and 6 months-old TgF344-AD and WT rats.** A) Distance matrices based on the results of the hierarchical clustering of all 200 extracted QPPs. Yellow colors indicate a high distance, while blue colors mean a small distance (high similarity). After applying all criteria to select robust clusters, only 1 cluster remained for each group at each time point. B) Statistically lower cluster wise occurrence rates for long QPPs at 4 months, but not at 6 months (two sample t-tests, FDR p<0.05)


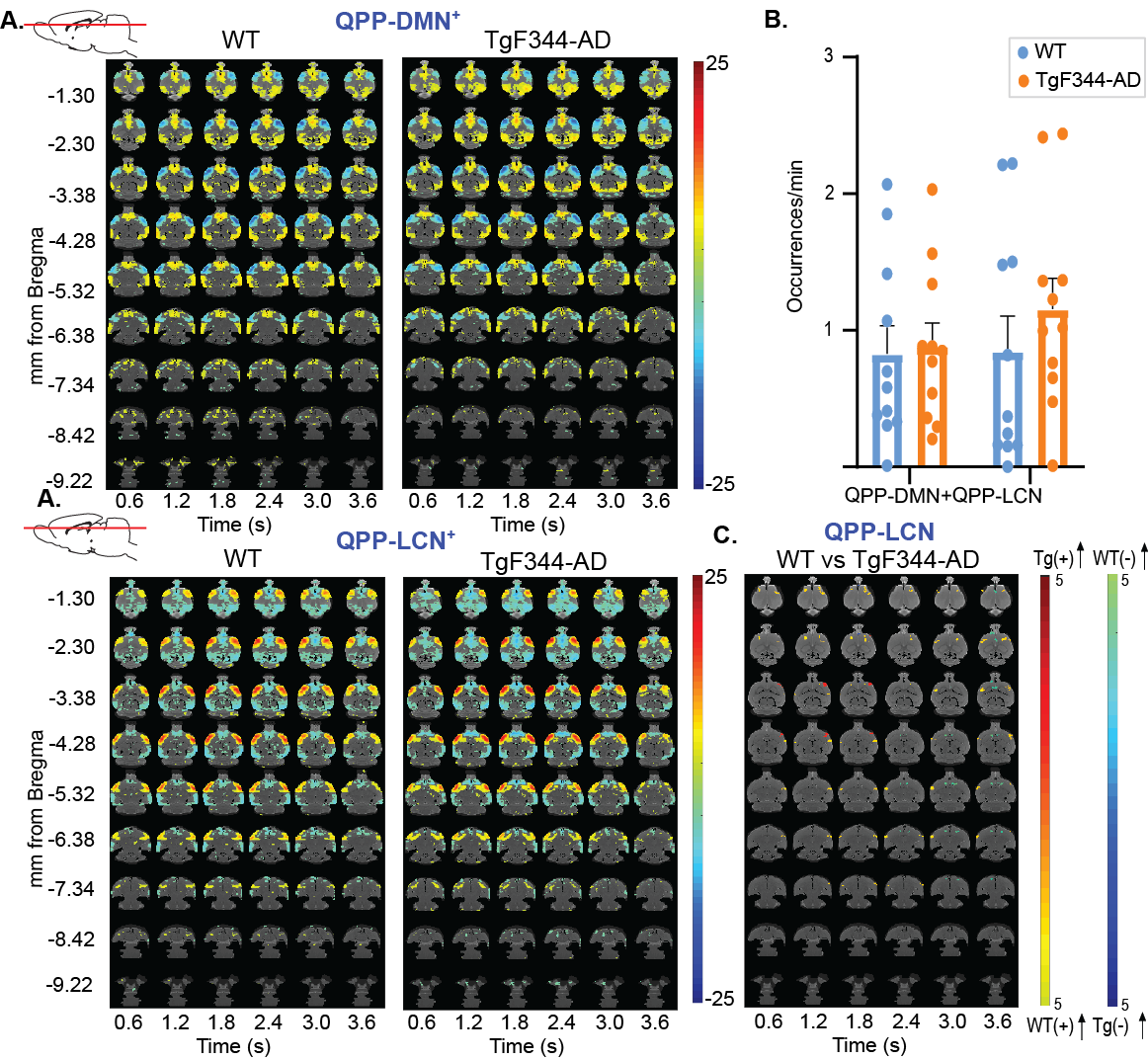


**Supplementary figure 6:** **Spatiotemporal alterations in network activity in 6-month old TgF344-AD rats (Tg) as compared to control littermates (WT).** A) T-maps (two-tailed one sample T-tests, FDR p<0.05, minimal cluster size 10) show the two rQPPs in each group. Comparison of cluster wise-occurrence rates between groups (two-sample t-test, two-tailed, FDR p<0.05) demonstrate no significant differences in occurrence of both QPPs between groups. C) T-maps (two-tailed two sample T-tests, FDR p<0.05, minimal cluster size 10) between rQPP-LCN. The warm colors (red/yellow) indicate a significant difference in BOLD activity between groups for voxels which are positive (activated) in the original QPP of the WT group. The right color bar (light blue/dark blue) indicate significant genotype differences for voxels which were deactivated in the original QPP of the WT group.


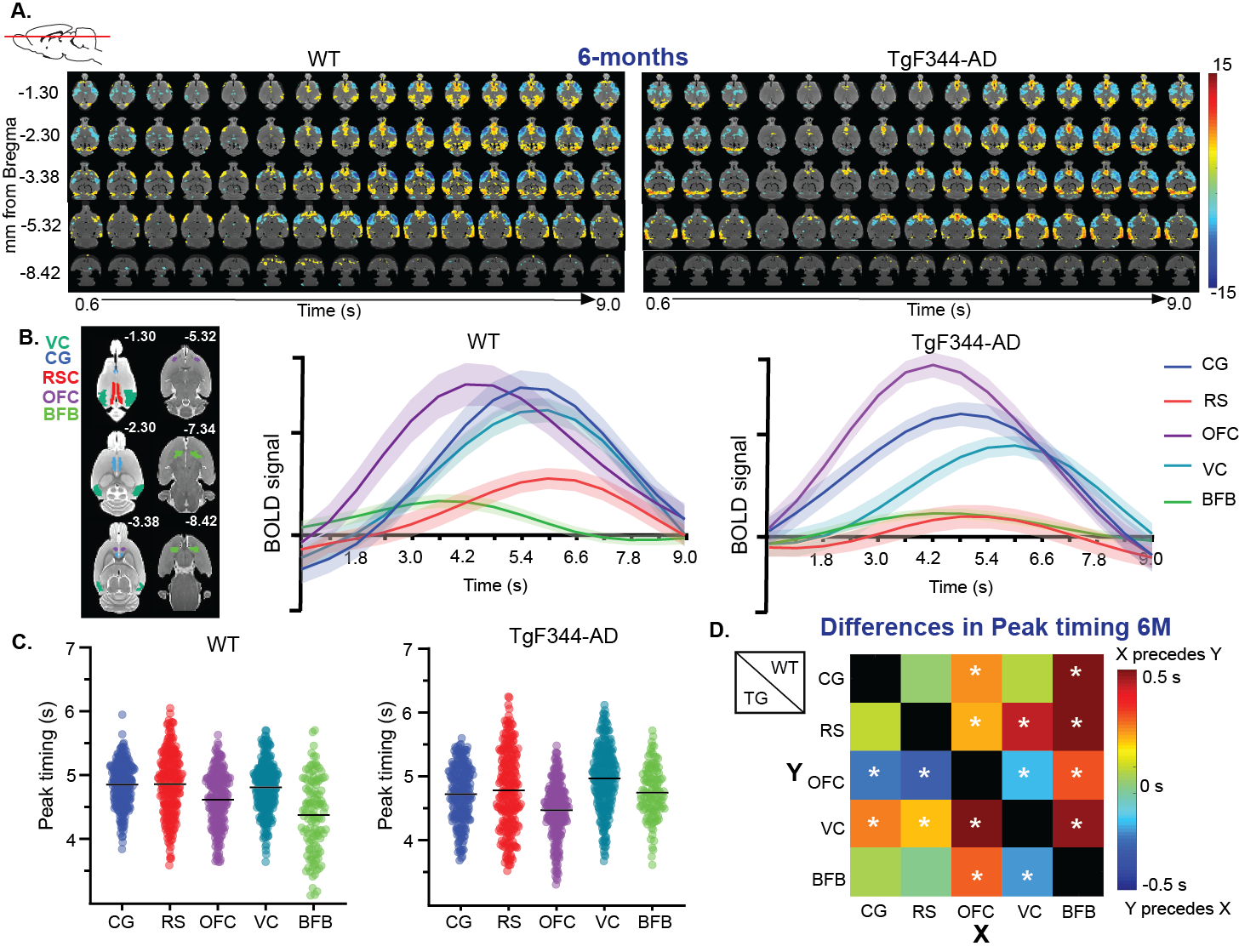


**Supplementary figure 7: Long QPPs to investigate propagation of network activity at the early-plaque stage** A) T-maps (two-tailed one sample t-tests, FDR p<0.05, minimal cluster size 10) show the long rQPPs in each group. Colors indicate T-values. B) average BOLD timecourses within regions of interest during 9-second QPPs. C) Peak timings averaged across occurrences for each voxel within a certain region. Dots represent the distribution of average peak timing of individual region of interest (ROI)-specific voxels; the black lines represent the mean peak timing across all ROI-specific voxels. D) Differences (peak_y_-peak_x_) in mean peak timing across voxels for each connection (X=row, Y=column). The top half represents the wildtype (WT) animals, while the lower half represents the TgF344-AD rats (Tg). Unpaired two-sample t-tests (FDR p<0.05) between regions were performed to evaluate if peak timing was significantly different between ROIs. CG = Cingulate cortex, OFC = orbitofrontal cortex, RSC = retrosplenial cortex, HC = hippocampus, BFB = basal forebrain. * p<0.05Supplementary tables

**Supplementary Table 1: p-values two-way ANOVA pTau**

| pTau | Age*Genotype | Genotype | Age |
| --- | --- | --- | --- |
| CG | 0,9699 | 0,8299 | 0,0016 |
| RS | 0,3607 | 0,8738 | 0,0109 |
| Ent | 0,9525 | 0,0559 | 0,0007 |
| SS | 0,2271 | 0,8903 | 0,0685 |
| CA1 | 0,3425 | 0,0066 | 0,0008 |
| DG | 0,9027 | 0,049 | 0,0537 |
| MS | 0,9137 | 0,6721 | 0,0019 |
| HDB/SI | 0,7068 | 0,6779 | 0,0004 |
| NBM | 0,5721 | 0,2408 | 0,001 |
| LC | 0,1473 | 0,0169 | 0,0596 |

Statistical analysis of the %area pTau between groups and ages. CG = cingulate cortex, RS = retrosplenial cortex, Ent = entorhinal cortex, SS = somatosensory cortex, DG = dentate gyrus, MS = medial septum, HDB/SI = horizontal limb of the diagonal band of Broca/substantia innominata, NBM = nucleus basalis of Meynert, LC = locus Coeruleus.

**Supplementary table 2**

| **4 Months** | | | **6 Months** | | |
| --- | --- | --- | --- | --- | --- |
| **Connection** | **NO FDR** | **FDR p<0.05** | **Connection** | **NO FDR** | **FDR p<0.05** |
| Cg L-CA3 L | 0.004572 | 0.310912516 | CG R - OFC R | 0.037731 | 0.996217 |
| Cg L-BFB L | 0.0428 | 0.452362018 | CG R - Limbic L | 0.007888 | 0.996217 |
| Cg R-DG R | 0.029925 | 0.441358644 | CG R - NAcc R | 0.023084 | 0.996217 |
| Cg R-BFB L | 0.021608 | 0.441358644 | RSC L - VC R | 0.019694 | 0.996217 |
| RSC L - DG L | 0.005966 | 0.310912516 | OFC L - Pir L | 0.029186 | 0.996217 |
| RSC L - MC L | 0.048716 | 0.482118744 | TeA R - S2 R | 0.039435 | 0.996217 |
| RSC L - MC R | 0.035527 | 0.441358644 | CA1 L - S1 R | 0.019032 | 0.996217 |
| RSC L - FrA R | 0.032318 | 0.441358644 | CA1 R - BFB R | 0.048509 | 0.996217 |
| RSC L - Ins R | 0.023681 | 0.441358644 | DG L - S2 R | 0.005793 | 0.996217 |
| RSC L - Thal R | 0.030434 | 0.441358644 | DG R - Aud R | 0.019306 | 0.996217 |
| RSC R - CA3 R | 0.037874 | 0.441358644 | DG R - FrA R | 0.038933 | 0.996217 |
| RSC R - S1 R | 0.039962 | 0.441358644 | DG R - NAcc R | 0.01786 | 0.996217 |
| OFC L - BFB L | 0.00664 | 0.310912516 | VC L - Thal L | 0.019745 | 0.996217 |
| OFC R - FrA R | 0.041174 | 0.443133724 | Aud L - S2 R | 0.006001 | 0.996217 |
| OFC R - BFB L | 0.00801 | 0.313462792 | Aud R - S1 R | 0.039051 | 0.996217 |
| Limbic L - Ent R | 0.002935 | 0.310912516 | Aud R - S2 R | 0.005965 | 0.996217 |
| Limbic L - VC R | 0.011572 | 0.357813665 | Pir L - BFB R | 0.023854 | 0.996217 |
| Limbic L - Aud R | 0.006022 | 0.310912516 | FrA L - Ins R | 0.033751 | 0.996217 |
| Limbic L - S2 L | 0.035254 | 0.441358644 | FrA L - Thal R | 0.0319 | 0.996217 |
| Limbic L - S2 R | 0.03868 | 0.441358644 | Cpu L - BFB L | 0.023623 | 0.996217 |
| **Limbic L - MC L** | **0.000897** | **0.257341465** | Pcu R - Thal R | 0.035732 | 0.996217 |
| Limbic L - CPu L | 0.030139 | 0.441358644 | NAcc R - BFB L | 0.041065 | 0.996217 |
| Limbic L - BFB L | 0.011194 | 0.357813665 | BFB R - Thal L | 0.025308 | 0.996217 |
| Limbic R - TeA L | 0.040339 | 0.441358644 |  |  |  |
| Limbic R - TeA R | 0.027054 | 0.441358644 |  |  |  |
| Limbic R - Ent R | 0.040496 | 0.441358644 |  |  |  |
| Limbic R - VC R | 0.007583 | 0.310912516 |  |  |  |
| Limbic R - Aud R | 0.037326 | 0.441358644 |  |  |  |
| Limbic R - S2 L | 0.006159 | 0.310912516 |  |  |  |
| Limbic R - MC L | 0.003935 | 0.310912516 |  |  |  |
| Limbic R - Nacc R | 0.038079 | 0.441358644 |  |  |  |
| TeA L - TeA R | 0.014949 | 0.396984614 |  |  |  |
| TeA L - DG R | 0.03066 | 0.441358644 |  |  |  |
| TeA L - Ent L | 0.027908 | 0.441358644 |  |  |  |
| TeA L - Aud R | 0.045215 | 0.469032389 |  |  |  |
| TeA R - Ent L | 0.003832 | 0.310912516 |  |  |  |
| TeA R - Aud R | 0.012823 | 0.368014375 |  |  |  |
| TeA R - Pir L | 0.020042 | 0.441358644 |  |  |  |
| TeA R - S1 L | 0.013564 | 0.376722946 |  |  |  |
| **TeA R - S1 R** | **0.000596** | **0.256527495** |  |  |  |
| TeA R - S2 L | 0.037873 | 0.441358644 |  |  |  |
| TeA R - S2 R | 0.015215 | 0.396984614 |  |  |  |
| TeA R - MC L | 0.038483 | 0.441358644 |  |  |  |
| CA1 L - DG R | 0.026123 | 0.441358644 |  |  |  |
| CA1 R - CA3 L | 0.004031 | 0.310912516 |  |  |  |
| CA3 L - S2 R | 0.006799 | 0.310912516 |  |  |  |
| CA3 R - S2 R | 0.038274 | 0.441358644 |  |  |  |
| CA3 R - Ins R | 0.030496 | 0.441358644 |  |  |  |
| DG L - S1 L | 0.029963 | 0.441358644 |  |  |  |
| DG L - S1 R | 0.003009 | 0.310912516 |  |  |  |
| DG R - Ent L | 0.031331 | 0.441358644 |  |  |  |
| DG R - Ins L | 0.006884 | 0.310912516 |  |  |  |
| DG R - Ins R | 0.005896 | 0.310912516 |  |  |  |
| Ent L - Ent R | 0.004227 | 0.310912516 |  |  |  |
| Ent L - VC L | 0.022572 | 0.441358644 |  |  |  |
| Ent L - Aud R | 0.011636 | 0.357813665 |  |  |  |
| Ent L - MC L | 0.024182 | 0.441358644 |  |  |  |
| Ent R - Aud R | 0.019545 | 0.441358644 |  |  |  |
| Ent R - Pir L | 0.049298 | 0.482337892 |  |  |  |
| VC L - MC L | 0.040352 | 0.441358644 |  |  |  |
| VC L - MC - R | 0.027543 | 0.441358644 |  |  |  |
| VC L - Thal L | 0.028635 | 0.441358644 |  |  |  |
| VC R - S2 R | 0.017624 | 0.433546875 |  |  |  |
| VC R - Ins L | 0.043082 | 0.452362018 |  |  |  |
| Aud L - S1 L | 0.03781 | 0.441358644 |  |  |  |
| Aud L - S2 L | 0.040204 | 0.441358644 |  |  |  |
| Aud R - S1 L | 0.035917 | 0.441358644 |  |  |  |
| Aud R - S1 R | 0.010543 | 0.357813665 |  |  |  |
| Aud R - S2 R | 0.027555 | 0.441358644 |  |  |  |
| Pir L - MC L | 0.019059 | 0.441358644 |  |  |  |
| Pir L - MC R | 0.002261 | 0.310912516 |  |  |  |
| Pir R - MC R | 0.008717 | 0.326314849 |  |  |  |
| Pir R - BFB L | 0.015962 | 0.404210954 |  |  |  |
| Pir R - BFB R | 0.037396 | 0.441358644 |  |  |  |
| S1L - S2 R | 0.012436 | 0.368014375 |  |  |  |
| **S1 L - MC L** | **0.000136** | **0.11678127** |  |  |  |
| S1L - NAcc R | 0.027633 | 0.441358644 |  |  |  |
| S1R - MC L | 0.011617 | 0.357813665 |  |  |  |
| S2 L - MC L | 0.037242 | 0.441358644 |  |  |  |
| S2 L - NAcc R | 0.026678 | 0.441358644 |  |  |  |
| S2 L - Thal R | 0.007319 | 0.310912516 |  |  |  |
| MC R - FrA R | 0.048438 | 0.482118744 |  |  |  |
| FrA L - FrA R | 0.046875 | 0.480467409 |  |  |  |
| FrA L - BFB R | 0.02425 | 0.441358644 |  |  |  |
| FrA R - Ins L | 0.033938 | 0.441358644 |  |  |  |
| CPu L - CPu R | 0.006175 | 0.310912516 |  |  |  |
| NAcc L - BFB L | 0.027758 | 0.441358644 |  |  |  |
| NAcc L - BFB R | 0.048705 | 0.482118744 |  |  |  |

P values of the statistically different connections in static FC between WT and TgF344-AD rats after uncorrected two-sample t-test before and after FDR correction.

**Supplementary Table 3: Effect sizes short QPPs**

|  | | Cg | RSC | BFB | VC | OFC-PrL |
| --- | --- | --- | --- | --- | --- | --- |
|  |  | **Cohens d** | **Cohens d** | **Cohens d** | **Cohens d** | **Cohens d** |
| 4M QPP DMLN | **0.6s** | 0.5307 | 0.3315 | 0.6159 | 0.0592 | 0.7650 |
|  | **1.2s** | 0.4961 | 0.3364 | 0.7635 | 0.2374 | 0.8311 |
|  | **1.8s** | 0.3975 | 0.2815 | 0.8289 | 0.3232 | 0.8347 |
|  | **2.4s** | 0.3192 | 0.2054 | 0.9898 | 0.3261 | 0.7843 |
|  | **3.0s** | 0.3095 | 0.1332 | 0.8546 | 0.2542 | 0.6928 |
|  | **3.6s** | 0.3657 | 0.0769 | 0.7435 | 0.0995 | 0.5605 |
|  | **Average** | **0.4031** | **0.2274** | **0.7993** | **0.2166** | **0.7447** |
| 4M QPP LCN | **0.6s** | 0.2561 | 0.0473 | 0.0773 | 0.1131 | 0.5217 |
|  | **1.2s** | 0.0236 | 0.1445 | 0.1889 | 0.3957 | 0.7992 |
|  | **1.8s** | 0.2102 | 0.3026 | 0.2424 | 0.5697 | 0.8506 |
|  | **2.4s** | 0.2985 | 0.3764 | 0.2213 | 0.5730 | 0.6479 |
|  | **3.0s** | 0.2472 | 0.3743 | 0.1467 | 0.4647 | 0.8381 |
|  | **3.6s** | 0.0779 | 0.3161 | 0.0537 | 0.2970 | 0.9669 |
|  | **Average** | **0.1856** | **0.2601** | **0.1550** | **0.4022** | **0.7707** |
| 6M QPP LCN | **0.6s** | 0.2038 | 0.0685 | 0.3812 | 0.2894 | 0.3290 |
|  | **1.2s** | 0.2005 | 0.0193 | 0.3881 | 0.2062 | 0.3799 |
|  | **1.8s** | 0.1545 | 0.0477 | 0.3588 | 0.0174 | 0.3907 |
|  | **2.4s** | 0.1051 | 0.1068 | 0.2835 | 0.2268 | 0.3561 |
|  | **3.0s** | 0.0811 | 0.1381 | 0.1731 | 0.4070 | 0.2796 |
|  | **3.6s** | 0.0978 | 0.1347 | 0.0552 | 0.3143 | 0.1956 |
|  | **Average** | **0.1405** | **0.0859** | **0.2733** | **0.2435** | **0.3218** |
| 6M QPP DMLN | **0.6s** | 0.1766 | 0.2181 | 0.0087 | 0.4699 | 0.2099 |
|  | **1.2s** | 0.1807 | 0.2908 | 0.0384 | 0.3341 | 0.1243 |
|  | **1.8s** | 0.1573 | 0.0926 | 0.0871 | 0.07677 | 0.0251 |
|  | **2.4s** | 0.1433 | 0.1282 | 0.1171 | 0.2086 | 0.0371 |
|  | **3.0s** | 0.1492 | 0.3155 | 0.1167 | 0.4509 | 0.0388 |
|  | **3.6s** | 0.1649 | 0.4150 | 0.0845 | 0.5973 | 0.0216 |
|  | **Average** | **0.1620** | **0.2434** | **0.0755** | **0.3563** | **0.0761** |

Effect sizes based on the voxel-averaged timecourses within each ROI. d= 0.1-0.2 very small effect size, d = 0.2-0.5 small effect size, d = 0.5-0.8 – medium effect size, d>0.8 = Large effect size

**Supplementary Table 4: p-values statistical comparison voxel-wise mean peak timings within groups**

|  | 4M | | | | 6M | | | |
| --- | --- | --- | --- | --- | --- | --- | --- | --- |
|  | WT | | TG | | WT | | TG | |
|  | Diff (s) | FDR  p<0,05 | Diff (s) | FDR  p<0,05 | Diff (s) | FDR p<0,05 | Diff (s) | FDR p<0,05 |
| CG-BFB | 0,4380 | <0,0001 | -0,1103 | 0,0036 | 0,5149 | <0,0001 | -0,0232 | 0,6230 |
| CG-OFC | 0,0384 | 0,3330 | 0,0877 | 0,0017 | 0,2237 | <0,0001 | 0,2518 | <0,0001 |
| CG-RS | -0,0288 | 0,5346 | 0,2286 | 0,4210 | -0,0056 | 0,8806 | -0,0587 | 0,3640 |
| CG-VC | -0,2658 | <0,0001 | -0,1205 | 0,0014 | 0,0442 | 0,1275 | -0,2455 | <0,0001 |
| RS-VC | -0,2370 | <0,0001 | 0,1438 | <0,0001 | 0,4235 | 0,123 | -0,1867 | 0,0014 |
| RS-OFC | 0,0672 | 0,1020 | 0,0684 | 0,02110 | 0,1901 | <0,0001 | 0,3102 | <0,0001 |
| RS-BFB | 0,4668 | <0,0001 | -0,0129 | 0,0004 | 0,5205 | <0,0001 | 0,0354 | 0,5590 |
| OFC-VC | -0,3042 | <0,0001 | 0,1991 | <0,0001 | -0,1901 | <0,0001 | -0,4968 | <0,0001 |
| OFC-BFB | 0,3996 | <0,0001 | -0,1980 | <0,0001 | 0,2912 | <0,0001 | -0,2748 | <0,0001 |
| VC-BFB | 0,7038 | <0,0001 | 0,01344 | 0,8440 | 0,4707 | <0,0001 | 0,222 | <0,0001 |

Differences (Diff) in mean voxel-wise peak timings between different regions in seconds in wildtype (WT) and TgF344-AD rats (TG). Significant difference in peak timings between regions was evaluated using a two-sample t-test with FDR correction (p < 0.05).

**Supplementary Table 5: p-values two-way ANOVA Astrogliosis**

| GFAP | Age*Genotype | Genotype | Age |
| --- | --- | --- | --- |
| CG | 0,3614 | 0,0724 | 0,9826 |
| RSC | 0,0936 | 0,0372 | 0,8474 |
| mENT | 0,3602 | 0,182 | 0,5808 |
| SS | 0,0657 | 0,6707 | 0,3552 |
| MS | 0,8643 | 0,8081 | 0,958 |
| NBM | 0,0581 | 0,0269 | 0,0027 |
| HDB/SI | 0,0262 | 0,0183 | 0,9064 |

Statistical analysis of the %area GFAP between groups and ages. CG = cingulate cortex, RS = retrosplenial cortex, Ent = entorhinal cortex, SS = somatosensory cortex, MS = medial septum, HDB/SI = horizontal limb of the diagonal band of Broca/substantia innominata, NBM = nucleus basalis of Meynert

**Supplementary Table 6: p-values two-way ANOVA Microgliosis**

| Iba-1 | Age*Genotype | Genotype | Age |
| --- | --- | --- | --- |
| CG | 0,1571 | 0,8388 | 0,3466 |
| RSC | 0,2556 | 0,37 | 0,3707 |
| Ent | 0,1241 | 0,0937 | 0,0003 |
| SS | 0,5429 | 0,6633 | 0,0001 |
| MS | 0,3092 | 0,6105 | 0,0529 |
| HDB/SI | 0,3118 | 0,1338 | 0,55 |
| NBM | 0,8146 | 0,2123 | 0,0001 |

Statistical analysis of the %area Iba-1 between groups and ages. CG = cingulate cortex, RS = retrosplenial cortex, Ent = entorhinal cortex, SS = somatosensory cortex, MS = medial septum, HDB/SI = horizontal limb of the diagonal band of Broca/substantia innominata, NBM = nucleus basalis of Meynert

**Supplementary table 7: p-values two-way ANOVA vGLUT/vGAT**

| vGLUT/vGAT | Age*Genotype | Genotype | Age |
| --- | --- | --- | --- |
| CG | 0,2649 | 0,0199 | 0,0879 |
| RSC | 0,3099 | 0,0283 | 0,0001 |
| Ent | 0,3686 | 0,1561 | 0,5679 |
| SS | 0,0548 | 0,0792 | 0,0469 |
| MS | 0,0232 | 0,7135 | 0,0001 |
| HDB/SI | 0,505 | 0,6998 | 0,95 |
| NBM | 0,7753 | 0,1531 | 0,0427 |

Statistical analysis of the ratio of glutamatergic and gaba-ergic synapses between groups and ages. CG = cingulate cortex, RS = retrosplenial cortex, Ent = entorhinal cortex, SS = somatosensory cortex, MS = medial septum, HDB/SI = horizontal limb of the diagonal band of Broca/substantia innominata, NBM = nucleus basalis of Meynert

**Supplementary table 8: p-values ANOVA cholinergic synaptic count**

| #Cholinergic | Age*Genotype | Genotype | Age |
| --- | --- | --- | --- |
| CG | 0,4059 | 0,8895 | 0,4253 |
| RSC | 0,2947 | 0,2502 | 0,6918 |
| Ent | 0,8684 | 0,9815 | 0,0402 |
| SS | 0,8174 | 0,9282 | 0,1037 |
| MS | 0,472 | 0,3097 | 0,336 |
| HDB/SI | 0,5189 | 0,7353 | 0,0001 |
| NBM | 0,1872 | 0,2538 | 0,5551 |

Statistical analysis of the number of cholinergic synapses between groups and ages. CG = cingulate cortex, RS = retrosplenial cortex, Ent = entorhinal cortex, SS = somatosensory cortex, MS = medial septum, HDB/SI = horizontal limb of the diagonal band of Broca/substantia innominata, NBM = nucleus basalis of Meynert
